# *Chlamydia trachomatis* exploits sphingolipid metabolic pathways during infection of phagocytes

**DOI:** 10.1128/mbio.03981-24

**Published:** 2025-04-18

**Authors:** Adriana Moldovan, Fabienne Wagner, Fabian Schumacher, Dominik Wigger, David Komla Kessie, Marcel Rühling, Kathrin Stelzner, Regina Tschertok, Louise Kersting, Julian Fink, Jürgen Seibel, Burkhard Kleuser, Thomas Rudel

**Affiliations:** 1 Department of Microbiology, University of Würzburg 9190, Würzburg, Bavaria, Germany; 2 Institute of Pharmacy, Freie Universität Berlin 54204, Berlin, Berlin, Germany; 3 Institute of Organic Chemistry, University of Würzburg 9190, Würzburg, Bavaria, Germany; Institut Pasteur, Paris, France

**Keywords:** sphingolipids, *Chlamydia*, macrophage, neutrophil, phagocyte, sphingosine kinase 1, sphingosine, sphingosine-1-phosphate phosphatase 2

## Abstract

*Chlamydiae* are obligate intracellular pathogens that utilize host cell metabolites for catabolic and anabolic processes. The bacteria replicate in epithelial cells from which they take up sphingolipids (SL) and incorporate them into the chlamydial membrane and the vacuole (termed *inclusion*). SL uptake is essential for *Chlamydia trachomatis* (*Ctr*) in epithelial cells; however, they can also infect phagocytes, but the consequences for the SL metabolism have not yet been investigated in these cells. We performed a quantitative sphingolipidome analysis of infected primary neutrophils, macrophages, and immortalized fallopian tube epithelial cells. Sphingosine (Sph) levels are elevated in primary M2-like macrophages and human neutrophils infected with *C. trachomatis*. Human neutrophils respond to the pathogen by markedly upregulating sphingosine kinase 1 (*SPHK1*). We show in M2-like macrophages, by RNAseq, that two counteracting pathways involving upregulation of *SPHK1*, but also sphingosine-1-phosphate phosphatases 1 and 2 (*SGPP1* and *SGPP2*) and sphingosine-1-phosphate lyase (*SGPL1*), maintain a steady pool of S1P. Using click chemistry, we show that exogenously added sphingomyelin (SM) and ceramide (Cer) are efficiently taken up into the chlamydial inclusion and are integrated into bacterial membranes in infected M2-like macrophages. Exogenous Sph reduces chlamydial infectivity, is transported into the inclusion lumen, and integrates into chlamydial membranes, suggesting that this particular SL species could represent a host defense mechanism. Taken together, our data indicate an important role for Sph/Sph kinase vs S1P/S1P phosphatase balance in infected phagocytes and a previously unrecognized role for sphingosine in the immune defense against chlamydial infection.

**IMPORTANCE:**

*Chlamydia trachomatis* (*Ctr*) is the leading cause of sexually transmitted diseases worldwide. Left untreated, it can cause severe complications such as blindness, pelvic inflammatory disease, or infertility. To date, no vaccines are available, and antibiotic treatment represents the only therapeutic approach to cure the infection. Limited access to antibiotics and displaced antibiotic intake increase the risk of developing recurring infections. Immune cells which fail to clear the infection and serve as a niche for chlamydial survival and replication, favor this outcome. Our research aims to elucidate the influence of sphingolipids (SL) during chlamydial infection, especially of phagocytic cells. Identifying relevant targets offers new strategies to develop alternative treatment methods.

## INTRODUCTION

According to the World Health Organization, 128.5 million new *Chlamydia trachomatis* (*Ctr*) infections were reported in 2020, making it the leading cause of sexually transmitted diseases worldwide (1). *Ctr* infections are commonly treated with antibiotics such as tetracycline or azithromycin; however, there is evidence that multidrug-resistant *Ctr* strains are emerging (2, 3). Urogenital infections can cause severe symptoms: ectopic pregnancy (4), pelvic inflammatory disease (5), or infertility (6). In most cases, *Ctr* infections remain asymptomatic, which favors the spread and the chronicity of the disease (7). The success of *Ctr* to establish persistent infections lies in its ability to evade the host cell immune response, especially the neutrophil response (8–10).

*Chlamydia* are obligate intracellular bacteria, with a biphasic life cycle. The *Ctr* infection cycle is initiated by the binding of elementary bodies (EB) to host cell surface receptors, followed by internalization mediated by host cell cytoskeleton remodeling, and concludes with the formation of a replicative compartment termed *inclusion* (11, 12). The replication cycle requires EB to reticulate body (RB) transition, followed by replication, RB to EB differentiation, and release of infectious EBs by host cell lysis or extrusion (13).

Blood circulating polymorphonuclear neutrophils (PMNs) are known to be recruited to the sites of microbial infection (14) in response to cytokines and chemokines produced by infected epithelial cells. There, they exert various antimicrobial functions, such as the release of reactive oxygen species, proteases, and antimicrobial peptides (15), as well as the formation of neutrophil extracellular traps (NETosis) (16, 17). However, these can be counteracted by chlamydial protease-like activating factor (CPAF), thereby supporting prolonged intracellular survival of *Ctr* within these phagocytic cells (9).

Within the genital tract, tissue-resident macrophages (18) are likely to encounter *Ctr* even earlier, upon the pathogen’s initial contact with the genital mucosa or upon egress from epithelial cells (10). In addition, circulating peripheral-blood mononuclear cells (PBMCs), which migrate into tissues (e.g., in response to inflammation) can differentiate into macrophages. Macrophages are specialized phagocytes, with crucial roles ranging from antimicrobial control to tissue homeostasis and wound repair (19). The diversity of functions performed by this cell type is largely dictated by their microenvironment (19, 20). A conventional approach to study them *in vitro* is to classify them as either classically activated (“M1”) or alternatively activated (“M2”) (21). In contrast to *“*M1” macrophages, which exhibit bactericidal functions, “M2” macrophages were shown to allow the growth of *Ctr* (22), as well as of *Chlamydia pneumoniae* (23) and *Chlamydia muridarum* (24).

Due to its reduced genome, *Ctr* depends on nutrient acquisition from the host cell (25), and therefore the bacterium interferes with host cell trafficking and recycling pathways (26–28). Among the hijacked metabolites, the sphingolipids (SLs), ceramide (Cer), and sphingomyelin (SM) are essential for *Ctr* replication, as well as for inclusion growth and stability (29, 30). SLs are structural components of biological membranes with some SL species acting as signaling molecules during various processes including phagocytosis (31) and apoptosis (32). Most importantly, several studies have attributed antimicrobial properties to SLs (33–36). This particularly applies to sphingosine (Sph), which was shown to be active against various bacterial pathogens (33) such as *Pseudomonas aeruginosa*, *Staphylococcus aureus, Neisseria gonorrhoeae*, and *Escherichia coli* (36–38).

This study investigates the role of SLs and their metabolic enzymes during chlamydial infection of human phagocytes. We show that *Ctr* induces host cell-type-specific changes in the sphingolipidome, with marked accumulation of Sph as well as Cer in M2-like macrophages. We observe a significant increase in transcripts and enzyme activity of sphingosine kinase 1 (*SPHK1*), one of two isoenzymes that convert Sph into sphingosine-1-phosphate (S1P), in response to *Ctr* infection. Despite the increase in SPHK activity, we do not detect elevated levels of S1P, which in M2-like macrophages is likely due to a simultaneous upregulation of S1P phosphatases (*SGPP1* and *SGPP2*) and S1P lyase (*SGPL1*). Pathogen-induced upregulation of the *SGPP2* phosphatase in M2-like macrophages has not been reported to our knowledge. Moreover, we observe that not only is exogenous Sph readily recruited to the chlamydial membrane, but Sph treatment also drastically reduces the infectivity of *Ctr* EBs.

## RESULTS

### *C. trachomatis* infection alters the sphingolipidome of phagocytic cells

PMNs, blood-derived monocytes, and macrophages, collectively termed professional phagocytes, were shown to be recruited to the site of chlamydial infection (14), supporting the notion that immune cells play crucial roles in the interaction with *Ctr*.

To investigate the role of SLs during *Ctr* infection in professional phagocytes, we used human primary blood monocyte-derived macrophages, polarized to either an alternatively activated “M2-like” or classically activated “M1-like” type (henceforth referred to as M2Φ and M1Φ, respectively), and primary peripheral blood-derived PMNs as targets for infection with the *lymphogranuloma venereum* L2 *Ctr* strain. For comparison, the fallopian tube epithelial cell line FT190 was also included in the analysis. Epithelial cells, as well as M2Φ, are known to be permissive for *Ctr* infection (22). While infection of epithelial cells is very efficient and *Ctr* establishes a stable replicative niche (~60% infection rate), in M2Φ, it is only ~8% of the cells that allow inclusion formation, *Ctr* replication, and infectious progeny production (Fig. S1).

**Fig 1:**
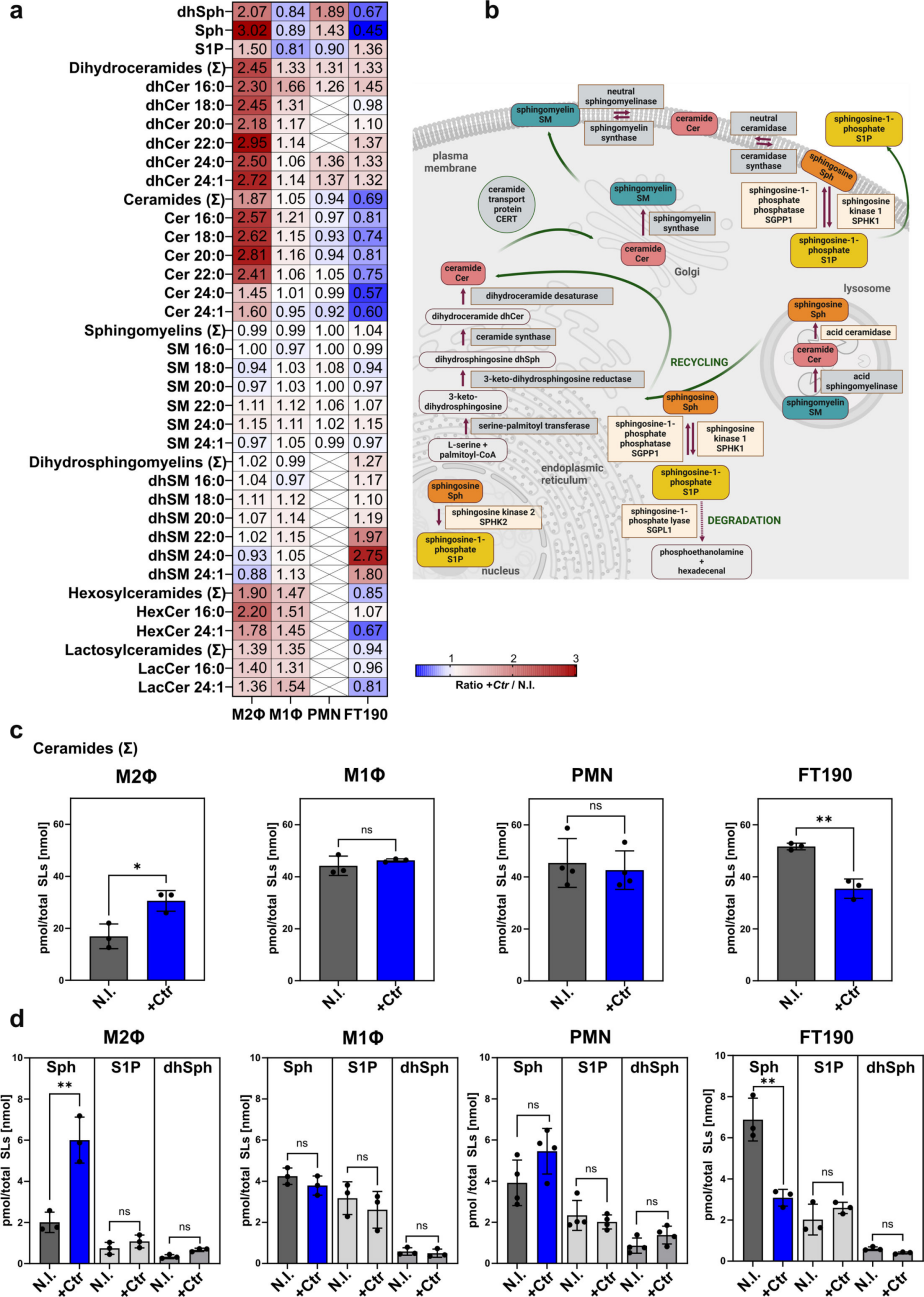
Infection with *C. trachomatis* alters sphingolipid levels in phagocytic cells. (a) Sphingolipidome of *Ctr*-infected vs uninfected cells. M2Φ, M1Φ, PMNs, and FT190 cells were infected with *Ctr* for 24 h (PMNs) or 30 h (M2Φ, M1Φ, FT190), or left uninfected (N.I.). After extraction, sphingolipids were analyzed by liquid chromatography tandem‐mass spectrometry (LC-MS/MS). Sphingolipid species were normalized to the total amount of sphingolipids. Heat map depicts sphingolipid profiles shown as a ratio of infected cells/non-infected cells (M2Φ and M1Φ: M2 or M1-like primary human macrophages; PMNs: primary human polymorphonuclear neutrophils; Sph: sphingosine; S1P: sphingosine-1-phosphate; dhSph: dihydrosphingosine). (b) Sphingolipid metabolism (adapted from reference 39). (c) Total ceramides in infected (*+*Ctr) and uninfected cells (N.I*.*). Data are shown as mean ± SD, from independent biological replicates (*n* ≥ 3). (d) Sphingoid long-chain bases in infected (*+*Ctr) and uninfected cells (N.I.). Data are shown as mean ± SD, from independent biological replicates (*n* ≥ 3). Data shown in panels c and d originate from the data set depicted in panel a. An unpaired, two-tailed *t*-test was used for analysis (ns, not significant; *, *P* < 0.05; **, *P* < 0.01).

At 24 h (for PMN) or 30 h (for M2Φ, M1Φ, and FT190) post-infection (p.i.), SL species were analyzed by targeted mass spectrometry. Our analysis indicated marked cell-type-specific differences in the abundance of individual SL species (Fig. 1a). A schematic overview of the SL species and their metabolic pathways is shown in Fig. 1b. M2Φ showed a very distinct pattern compared to all other cell types, whereby all species of the *de novo* synthesis pathway were enriched. Interestingly, total Cer levels were significantly increased in M2Φ and decreased in FT190, with no drastic changes in either M1Φ or PMN (Fig. 1c). In addition, we found Sph significantly increased in M2Φ upon infection, with a similar trend in PMNs (Fig. 1d). In contrast, in FT190 cells, *Ctr* infection caused a significant decrease in Sph levels, whereas in M1Φ they remained unaltered (Fig. 1d). S1P levels were not significantly altered in any of the investigated cell types (Fig. 1d).

Taken together, our data reveal host cell-type-specific changes in SL levels upon chlamydial infection. The elevated Sph levels observed in professional phagocytes, but not in epithelial cells, suggest a role for Sph during the immune response.

### Sphingosine kinases are upregulated during infection of phagocytes with *C. trachomatis*

**Fig 2:**
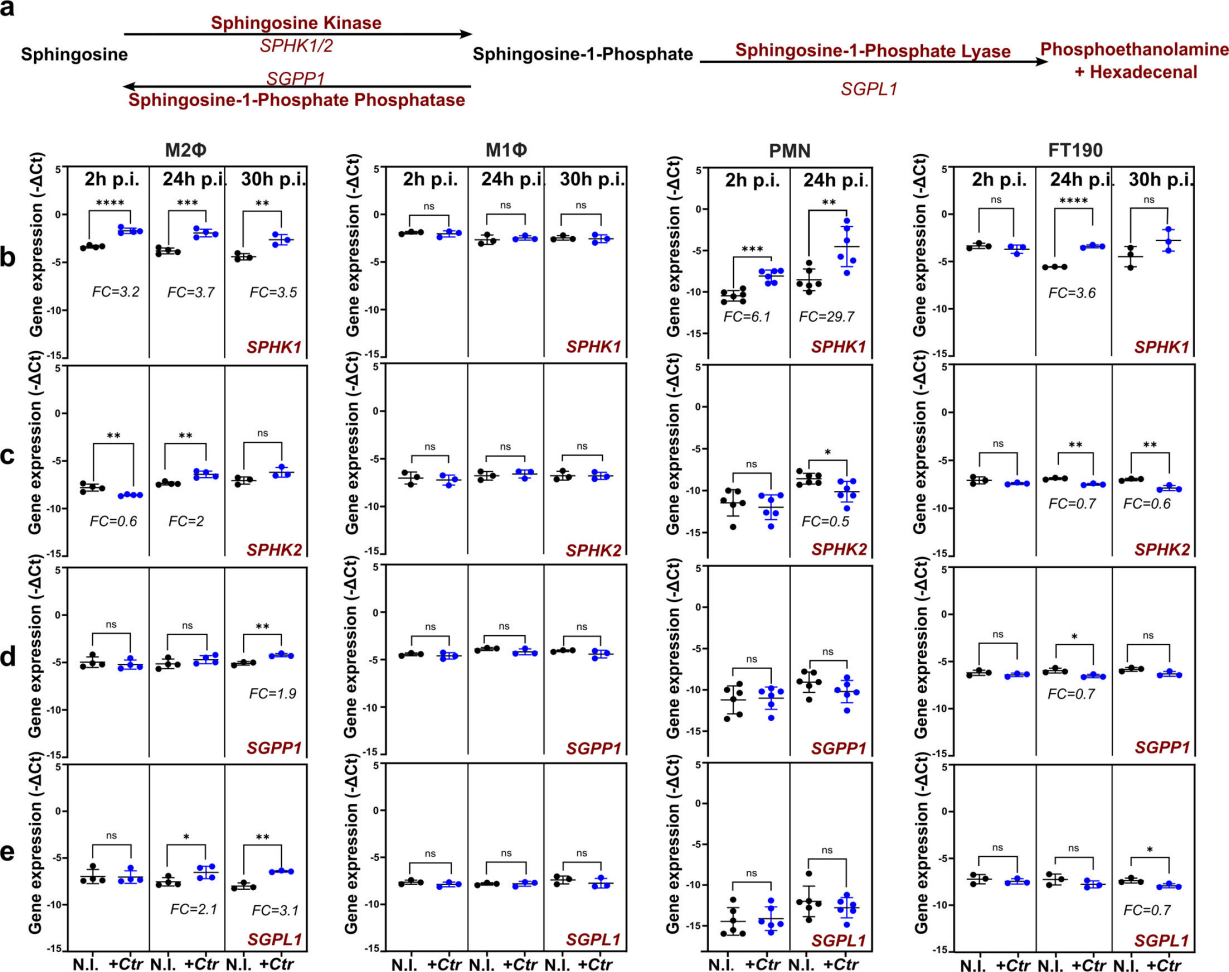
Expression of sphingolipid metabolic genes is altered upon *C. trachomatis* infection. (a) Schematic representation of the sphingolipid metabolism (adapted from reference 39). Sph is converted to S1P in a reversible process by the action of sphingosine kinases and S1P phosphatases. To permanently remove S1P from the SL pool, it is broken down to phosphoethanolamine and hexadecenal by the S1P lyase. (**b**–e) Gene expression profiles of sphingolipid metabolic genes. Sphingosine kinase 1 and 2 (*SPHK1/2*), S1P phosphatase 1 (*SGPP1*), and S1P lyase 1 (*SGPL1*) were investigated. M2Φ, M1Φ, PMN, and FT190 cells were left uninfected (N.I.) or were infected with *Ctr* (*+Ctr*) for 2, 24, and 30 h. Gene expression is shown as *−*Δ*Ct*. Fold change (FC) is indicated and expressed as 2^−ΔΔCt^ (infected vs uninfected). Data are shown as mean ± SD, from independent biological replicates (*n* ≥ 3). An unpaired, two-tailed *t*-test was used for analysis (ns, not significant; *P < 0.05; **P < 0.01; ***P ≤ 0.001; ****P ≤ 0.0001). M2Φ, M1Φ: M2 or M1-like primary human macrophages; PMNs: primary human polymorphonuclear neutrophils; p.i.: postinfection.

As infection with *Ctr* resulted in an increase in Sph levels in both PMNs and M2Φ, while S1P levels did not significantly change (Fig. 1d), we next sought to investigate whether alterations in the expression of genes encoding relevant enzymes in the sphingolipid metabolism (Fig. 2a) could account for the observed phenotypes. Gene expression was analyzed by quantitative reverse transcription-polymerase chain reaction (RT-qPCR) in M2Φ, M1Φ, PMN, and FT190 cells at the indicated time points after *Ctr* infection.

SPHK1/2 phosphorylate Sph to generate S1P (39). Since our sphingolipidome profiling revealed increased Sph levels, but not S1P, upon *Ctr* infection in M2Φ and PMNs, we were surprised to measure significantly increased transcript levels for *SPHK1* in both cell types, as early as 2 h p.i., as well as in FT190 cells 24 h p.i. (Fig. 2b). *SPHK2* expression was modestly regulated upon *Ctr* infection in M2Φ, with a twofold upregulation 24 h p.i. (Fig. 2c).

In mammalian cells, Sph and S1P are in a dynamic equilibrium with each other due to the opposing activities of the SPHK1/2 Sph kinases and the SGPP1 S1P phosphatase (39). Therefore, we next tested whether an additional upregulation of *SGPP1* could be responsible for maintaining S1P levels unaltered by counteracting the effects of *SPHK1* upregulation upon *Ctr* infection. Indeed, we observed a 1.9-fold increase in *SGPP1* expression 30 h p.i. in M2Φ, with little or no change in either PMNs or FT190 (Fig. 2d).

Another mechanism to control S1P levels is its permanent removal from the SL pool by degradation via the SGPL1 S1P lyase (39). Again, we found that *SGPL1* was upregulated in M2Φ 24 h and 30 h p.i. (Fig. 2e).

Notably, the expression of all investigated genes remained unaltered in M1Φ upon infection with *Ctr* (Fig. 2b through e).

Taken together, our results suggest that divergent SL metabolic pathways are employed by different cell types upon their interaction with *Ctr*. Whereas, in PMNs the response to the *Ctr* infection is characterized by a strong upregulation of *SPHK1,* at both early (2 h) and late (24 h) infection time points, in M2Φ two counteracting pathways involving upregulation of *SPHK1* as well as *SGPP1* and *SGPL1* likely maintain a steady pool of S1P.

### Sphingosine kinase activity increases during infection of phagocytes with *C. trachomatis*

**Fig 3:**
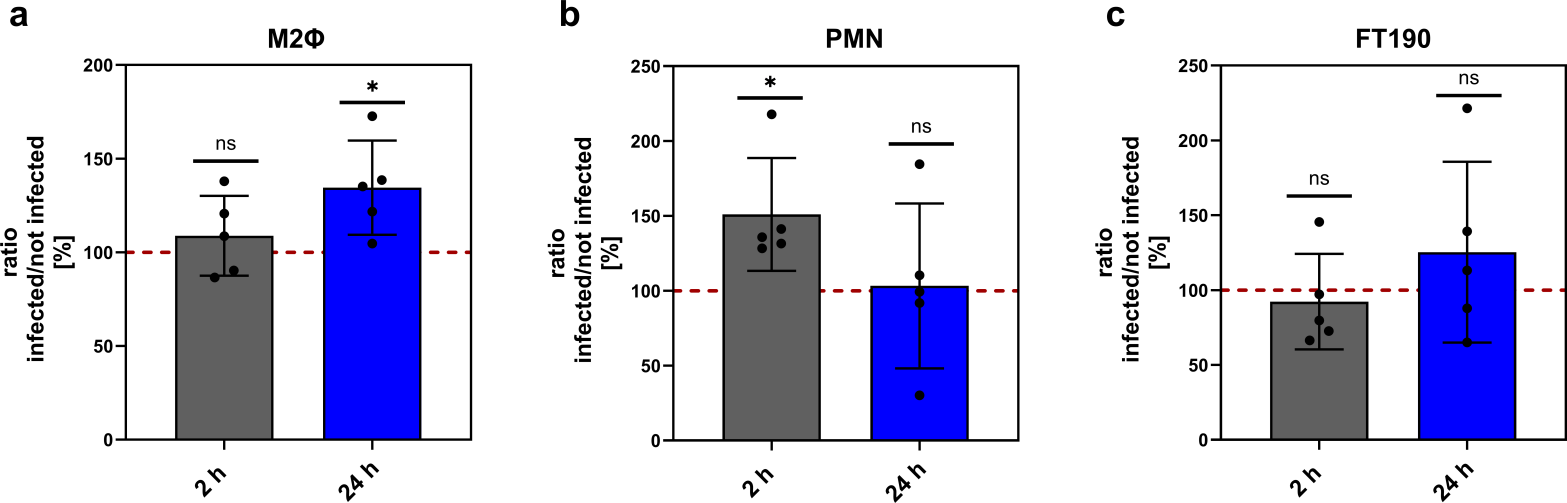
Sphingosine kinase activity is elevated upon *C. trachomatis* infection. Sphingosine kinase activity was measured for *Ctr*-infected and not infected M2Φ (a), PMN (b), and FT190 cells (c) at 2 and 24 h p.i. SPHK activity was measured via an ATP depletion assay. Results are shown as the ratio of infected cells divided by uninfected cells. The value of uninfected cells is set to 100%, marked as a dashed line. Data are shown as mean ± SD, *n* = 3. One sample *t*-test was used for analysis (ns, not significant; *P < 0.05). M2Φ: M2-like primary human macrophages; PMNs: primary human polymorphonuclear neutrophils.

Following up on the observation that *SPHK1* transcript levels increase after chlamydial infection of professional phagocytes, we next tested the enzymatic activity of SPHK. We performed SPHK activity assays 2 and 24 h after infection of M2Φ, PMN, and FT190 cells with *Ctr*. SPHK activity increased after *Ctr* infection in professional phagocytes at different time points. M2Φ showed a significantly increased SPHK activity only at the later infection time point (24 h p.i.) (Fig. 3a). On the other hand, in PMNs, SPHK activity drastically and transiently increased 2 h p.i., but at 24 h p.i., the activity was comparable to the uninfected controls (Fig. 3b). In FT190 cells, no significant differences were observed at any time point; however, a tendency toward increased activity 24 h p.i. was apparent (Fig. 3c).

### Transcriptional remodeling of sphingolipid metabolic pathways upon *C. trachomatis* infection of M2Φ

**Fig 4:**
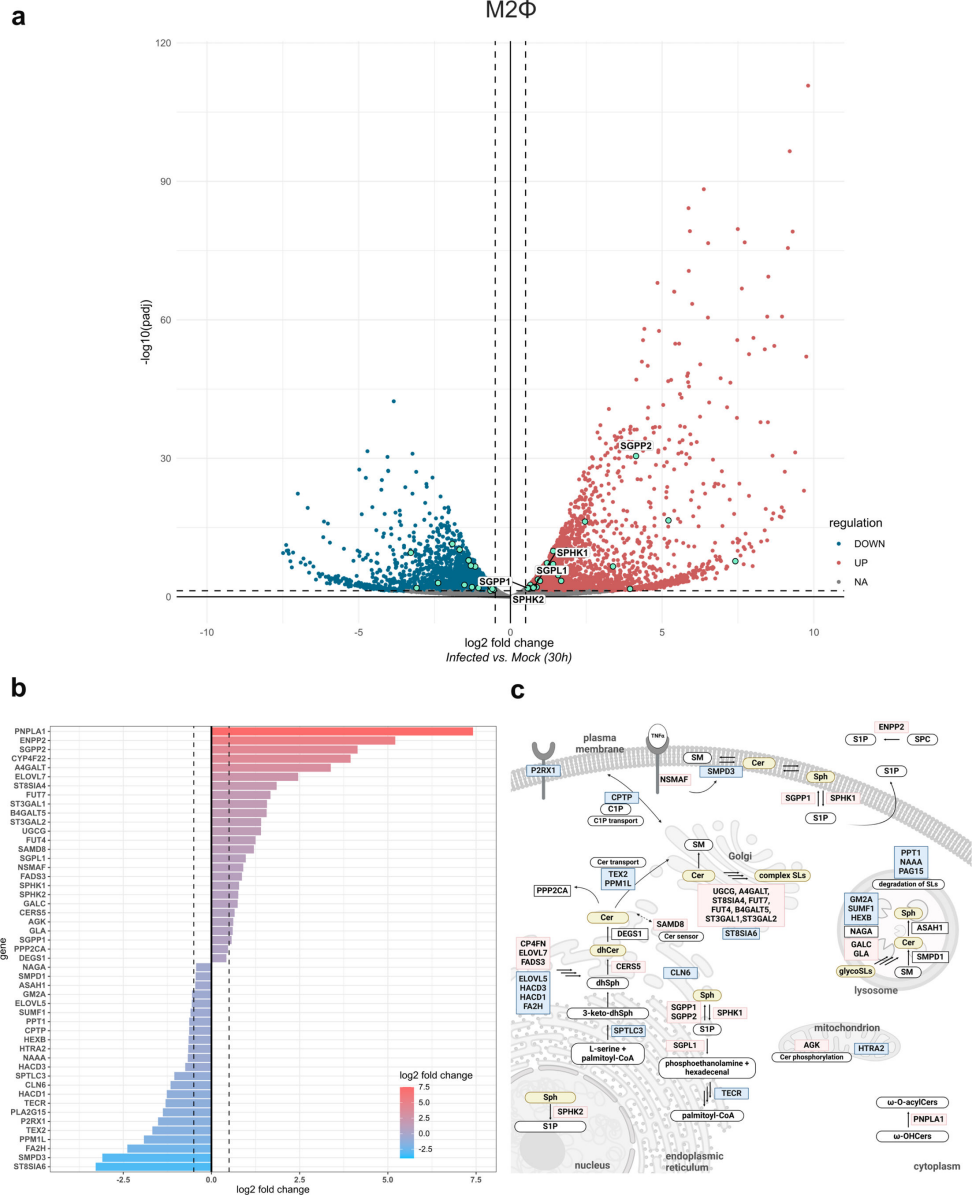
Transcriptional changes upon *C. trachomatis* infection in M2Φ macrophages. (a) Differential transcript abundance is expressed as log2 fold change (infected vs Mock) and plotted against −log10 adjusted *P*-values. Vertical lines represent the cutoff value for regulated genes (log2 fold change > 0.5 or < −0.5). The horizontal line represents the cutoff for *P*-values (adj. *P* < 0.05). Significantly upregulated genes are marked in red and downregulated genes are marked in blue. Significantly regulated genes associated with SL metabolism (annotated in GO:0006665 “sphingolipid metabolic processes” [40, 41]) are highlighted in green. (b) Transcriptional regulation of genes associated with SL metabolism 30 h p.i. log2 fold changes (infected vs Mock, 30 h p.i.) of 49 significantly changed genes (adj. *P* < 0.05, regardless of log2 fold change values) associated with SL metabolism (GO:0006665 [40, 41]) are plotted. Among the 49 genes, 44 were considered regulated (log2 fold change > 0.5 or < −0.5). Vertical lines indicate log2 fold change cutoff values (>0.5 or <−0.5). (c) Overview of sphingolipid metabolism (adapted from reference 39). SL metabolism-related genes which are significantly up (red) or downregulated (blue) upon *Ctr* infection (adj. *P* < 0.05 and log2 fold change > 0.5 or < −0.5) are highlighted. Lipid species found to be altered upon *Ctr* infection (Fig. 1a) are highlighted in yellow. Data are derived from three independent experiments (*n* = 3). Information was retrieved from UniProt (42).

The regulation of genes encoding enzymes relevant in the Sph/S1P axis prompted us to further question whether other pathways in the SL metabolism would be transcriptionally regulated upon *Ctr* infection. To this end, we performed RNAseq of *Ctr*-infected M2Φ at 30 h p.i. Among the 23,586 detected host transcripts, 3,514 genes were significantly upregulated and 3,160 were significantly downregulated upon infection (adj. *P* < 0.05, log2 fold change > 0.5, or < −0.5) (Fig. 4a).

Not only did the RNAseq experiment confirm our RT-qPCR results (i.e., upregulation of *SPHK1* and *SPHK2* but also *SGPL1* and *SGPP1*) (Fig. 4a; Data S1), but also revealed regulation of additional SL metabolism genes upon infection (Fig. 4; Fig. S2), in addition to a vast array of inflammatory mediators.

Among the genes involved in various SL metabolism processes including biosynthesis, catabolism, signaling, or transfer activity, ~30% were significantly regulated (Fig. 4b; Fig. S2; Data S1). For example, among others, *PNPLA1* (omega-hydroxyceramide transacylase), *ENPP2* (autotaxin/secreted lysophospholipase D), *A4GALT* (alpha 1,4-galactosyltransferase), *ELOVL7* (fatty acid elongase 7), *FADS3* (fatty acid desaturase 3), *CERS5* (ceramide synthase 5), or *NSMAF* (neutral sphingomyelinase activation associated factor) were upregulated (Fig. 4a and b; Data S1). Most surprisingly, *SGPP2*, encoding a second S1P phosphatase was markedly upregulated upon *Ctr* infection (log2 fold change = 4.1). An overview of pathways we found regulated upon infection of M2Φ (i.e., lipid species abundance and transcriptional regulation) is shown in Fig. 4c.

### *C. trachomatis* preferentially integrates metabolized rather than native SM into the inclusion

Functionalized SLs in combination with fourfold expansion microscopy (4× ExM) are a powerful tool to investigate sphingolipid distribution in infected cells. We have previously observed that α-amino-ω-azido-modified C6-ceramide can be incorporated both into the outer and inner chlamydial membrane in infected HeLa229 cells (43). Similarly, we now observed the integration of α-amino-ω-azido-C6-ceramide into the chlamydial membrane in infected M2Φ 30 h p.i. (Fig. S3). The ceramide transfer protein (CERT) has previously been shown to be recruited to the chlamydial inclusion in epithelial cells where it was suggested to function in the formation of ER-inclusion membrane contact sites (MCSs) that facilitate the transfer of SLs from the ER to the inclusion (44, 45). Similarly, we detected CERT in the inclusion membranes in *Ctr*-infected M2Φ (Fig. S3), suggesting that *Ctr* could exploit CERT for SL acquisition in M2Φ.

**Fig 5:**
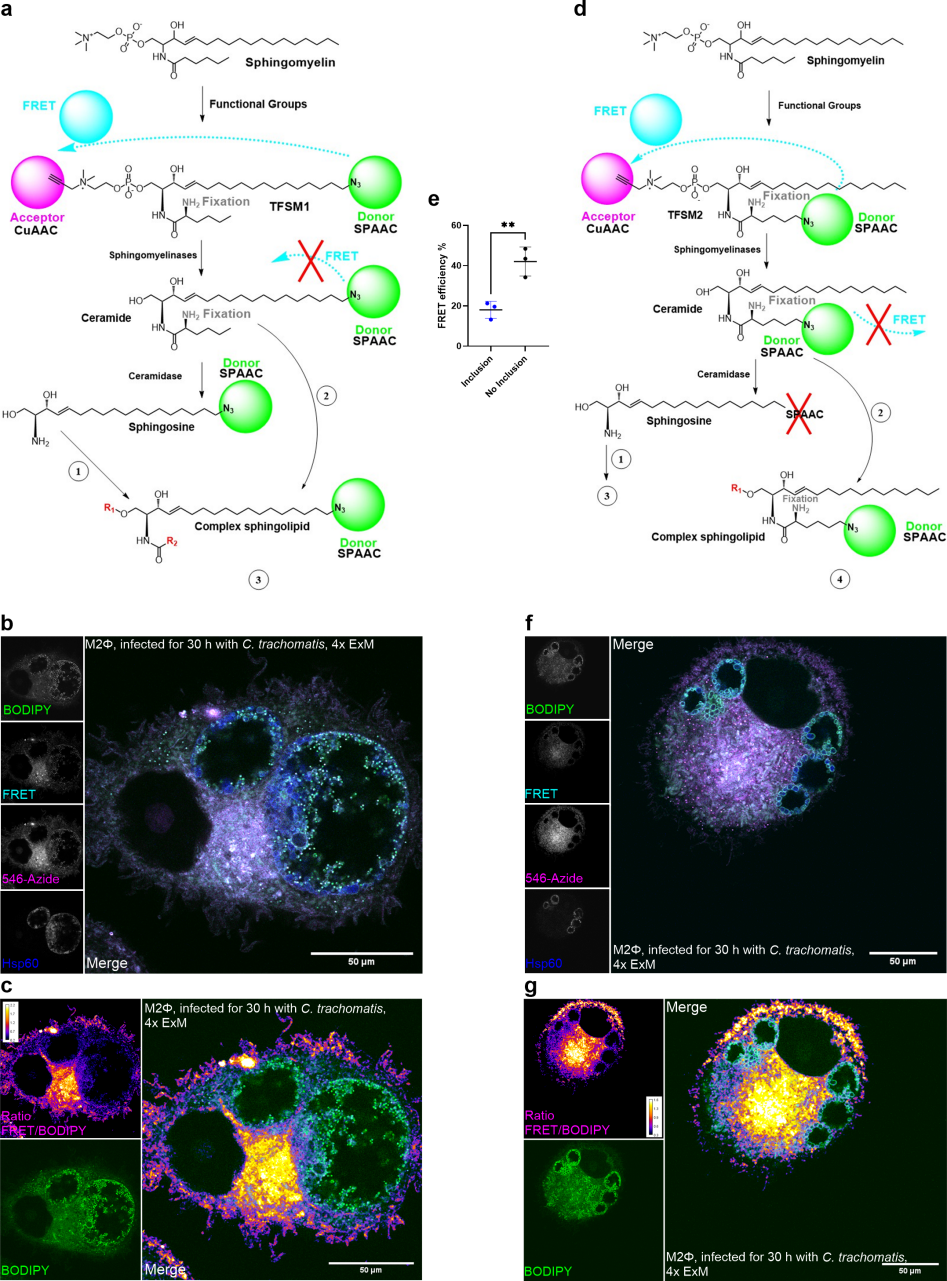
*C. trachomatis* preferentially incorporates metabolized sphingomyelin into the inclusion. (a) Schematic overview of TFSM1 metabolization. Enzymatic reaction 1 can be catalyzed by ceramide synthases, ceramide kinase, sphingomyelin synthases, and others. Reaction 2 can be carried out by ceramide kinase, sphingomyelin synthases, and others. Reaction 3: further metabolization of TFSM1 that maintains the sphingoid backbone results in a fluorescent labeled sphingolipid after SPAAC. (b) Confocal fluorescence image of a 4× expanded M2Φ infected with *Ctr* (30 h p.i.). 10 µM TFSM1 was added 27 h prior to fixation. The azido group was stained with BODIPY-FL-DBCO via SPAAC click chemistry (green), the alkyne group was stained with AlexaFluor 546 azide via CuAAC click chemistry (magenta), and *Ctr* was stained against Hsp60 (blue). The FRET channel is shown in cyan. Scale bar 50 µm (with 4-fold expansion factor ~12.5 µm). (c) The metabolic state of TFSM1 was calculated by dividing the signal of the FRET channel by the signal of the BODIPY-DBCO channel. The calibration bar displays the calculated ratio. (d) Schematic overview of TFSM2 metabolization. Enzymatic reaction 1 can be catalyzed by ceramide synthases, ceramide kinase, sphingomyelin synthases, and others. Reaction 2 can be carried out by ceramide kinase, sphingomyelin synthases, and others. Reaction 3: further metabolites of sphingosine derived from TFSM2 do not contain the acyl chain, thus no functional group, which means there are no fluorescent labeled sphingolipids after SPAAC. Reaction 4: further metabolization of TFSM2 that maintains the acyl chain results in a fluorescent labeled sphingolipid after SPAAC. (e) FRET efficiency was analyzed with the FRET AB wizard of the Leica TCS SP5 microscope. Acceptor bleaching was performed either at an area containing an inclusion or outside of the inclusion. Data are shown as mean ± SD from independent biological replicates (*n* = 3). One sample *t*-test was used for analysis (**P < 0.01). (f) Confocal fluorescence image of a 4× expanded M2Φ infected with *Ctr* (30 h p.i.). 10 µM TFSM2 was added 27 h prior to fixation. The azido group was stained with BODIPY-FL-DBCO via SPAAC click chemistry (green), the alkyne group was stained with AlexaFluor 546 azide via CuAAC click chemistry (magenta), and *Ctr* was stained against Hsp60 (blue). The FRET channel is shown in cyan. Scale bar 50 µm (with 4-fold expansion factor ~12.5 µm). (g) The metabolic state of TFSM2 was calculated by dividing the signal of the FRET channel by the signal of the BODIPY-DBCO channel. The calibration bar displays the calculated ratio. CuAAC: copper-catalyzed azide-alkyne cycloaddition, FRET: Förster resonance energy transfer; Hsp60: heat shock protein 60; M2Φ: M2-like primary human macrophages; SM: sphingomyelin; SPAAC: strain promoted azide–alkyne cycloaddition, TFSM: trifunctional sphingomyelin; 4x ExM: 4-fold expansion microscopy.

To further investigate SLs and their metabolization within M2Φ, we next used trifunctional SMs (TFSMs; Fig. 5). TFSMs (46) are functionalized SMs equipped with a primary amine group (for fixation with aldehyde fixatives) and two functional groups for click chemistry. The phosphocholine headgroup is modified with a terminal alkyne (for copper-catalyzed azide-alkyne cycloaddition, CuAAC), while the azide group is used for strain-promoted azide–alkyne cycloaddition (SPAAC). TFSM 1 (Fig. 5a) contains the azide in the sphingoid backbone, whereas TFSM2 (Fig. 5d) contains the azide in the acyl chain.

Using a Förster resonance energy transfer (FRET) pair for click chemistry, native SM emits a FRET signal, enabling the localization of SM. Upon cleavage by SMases, the functional headgroup is removed, and the FRET system is disabled, allowing visualization of SM degradation at nanoscale resolution. If TFSM1 is further metabolized, the azide group in the sphingoid backbone allows visualization of all SLs with intact sphingoid backbone: Cer, Sph, or S1P. TFSM2 only allows visualization of SLs that retain the acyl chain: Cer, ceramide-1-phosphate (C1P), or complex SLs. If the acyl chain is removed (e.g., upon generation of Sph), the functional group is lost, and visualization is no longer possible. We have recently shown that in epithelial cells, TFSMs are present inside the chlamydial inclusion preferentially in their metabolized form, rather than in the native form (46). Since SM levels were not altered in M2Φ upon chlamydial infection (Fig. 1a), we used the two TFSMs to evaluate the metabolic state of SM in *Ctr*-infected M2Φ 30 h p.i. We observed that chlamydial inclusions largely carried the metabolized form of both TFSMs (Fig. 5b, c, f, and g), as shown by the ratio of the FRET/BODIPY signal (Fig. 5c and g). The cellular structures of M2Φ appear as a bright yellow to magenta signal, indicating a higher ratio and thus a high proportion of FRET signal, i.e., the native molecule, in these areas. In contrast, chlamydial inclusions appear dark purple or blue. This indicates a lower ratio and thus a higher proportion of BODIPY signal compared to the FRET signal in these areas, implying that the signal is derived from metabolized TFSM. We validated this observation by FRET measurement of TFSM1 (Fig. 5e). We performed acceptor (AlexaFluor 546) bleaching either at an area containing an inclusion or outside of the inclusion. If the donor (BODIPY-FL) signal increases after acceptor bleaching, it means that a FRET pair was present at the bleached area. If the donor signal does not increase, there is no FRET pair present in this area. Thus, areas with a high FRET pair content show a higher FRET efficiency than areas with no FRET pairs. Measurement of FRET efficiency confirmed that inclusions showed 50% less FRET efficiency than areas outside the inclusion.

Metabolization of TFSM1 results in fluorescent SL species after SPAAC as long as the sphingoid backbone remains intact. Thus, TFSM1 does not allow differentiation of the different SL species inside the inclusion. TFSM2, on the other hand, loses the azide group after cleavage by ceramidases, so that once Sph is formed, SPAAC is no longer possible, and Sph, and other metabolites derived from it, cannot be visualized. Cer and all other complex SLs metabolized from TFSM2 still contain the acyl chain and can therefore be visualized with SPAAC. Using TFSM2, *Ctr* membranes inside M2Φ carried a BODIPY signal (Fig. 5f and g). Therefore, we hypothesize that the metabolized form of TFSM inside the chlamydial inclusions still contains the acyl chain and thus is metabolized to Cer or complex SLs rather than Sph. We cannot exclude the possibility that some of the metabolized Sph from TFSM2 is still present inside the inclusion, as it is not visualizable with this tool set. Taken together, we observe that exogenously added TFSM is present in a metabolized state inside the inclusion. However, using TFSMs in combination with 4× ExM only allows us to visualize metabolization *per se*, but not the identification of the resulting species. Furthermore, the preference of metabolized SM (e.g., Cer or other complex SL such as hexosylCer) over SM in the chlamydial inclusion is in agreement with our mass spectrometry data showing an increase in Cer but a constant level of SM in infected M2Φ (Fig. 1a).

### Sphingosine reduces the infectivity of chlamydial elementary bodies

**Fig 6:**
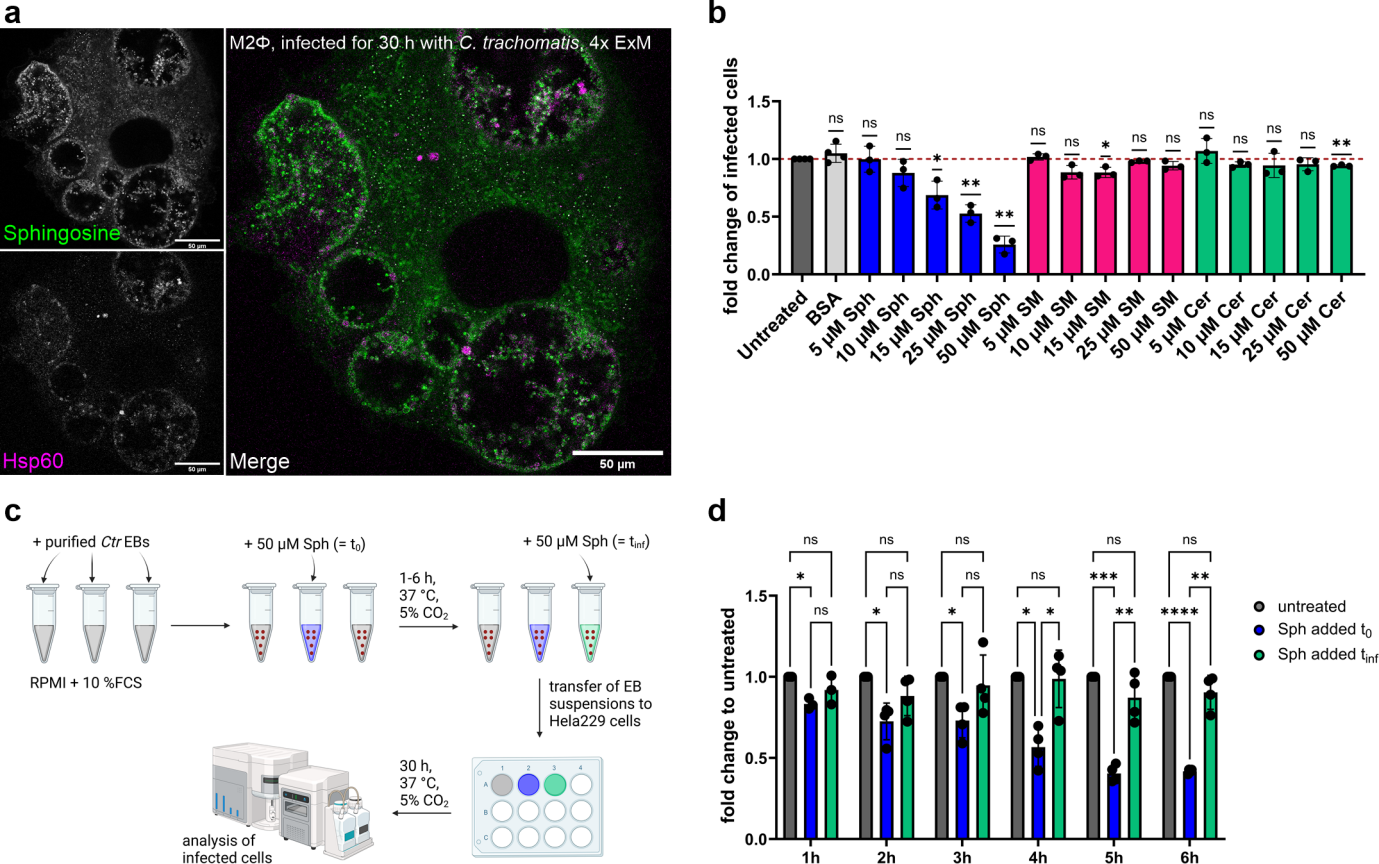
Sphingosine reduces infectivity of chlamydial elementary bodies. (a) Confocal fluorescence image of a 4× expanded M2Φ infected with *Ctr* (30 h p.i.). 10 µM ω-azido-sphingosine was added 1 h prior to fixation. Sph was stained with DBCO-AF488 via SPAAC click chemistry (green), and *Ctr* was stained against Hsp60 (magenta). Scale bar 50 µm (with 4-fold expansion factor ~12.5 µm). (b) mCherry-expressing chlamydial EBs were treated with the indicated amount of SL for 5 h and subsequently transferred to freshly plated HeLa 229 cells. After 30 h p.i., the number of infected HeLa 229 cells was analyzed by flow cytometry. Data are shown as fold change and are normalized to the untreated control sample, which is set to 1 and shown as a dashed line. Data are shown as mean ± SD, from independent biological replicates (*n* ≥ 3). One sample *t*-test was used for analysis (ns, not significant; *P < 0.05; **P < 0.01). (c) Workflow: mCherry expressing chlamydial EBs were left untreated or were treated with 50 µM Sph (Sph added at *t*_0_) for the indicated time and subsequently transferred to freshly plated HeLa 229 cells. One set of EBs was left untreated, but upon transfer to the cells, the respective amount of Sph was added to match the final concentration of Sph (Sph added *t*_inf_). After 30 h p.i. the number of infected HeLa 229 cells was analysed by flow cytometry. (d) The results of the experiment described in (c) are depicted. After 30 h p.i. the number of infected HeLa 229 cells was analyzed by flow cytometry. Data are shown as fold change and was normalized to the untreated control sample. Data are shown as mean ± SD, from independent biological replicates (*n* ≥ 3). Mixed-effects analysis was used for analysis (ns, not significant; *P < 0.05; **P < 0.01; ***P ≤ 0.001; ****P ≤ 0.0001). BSA, bovine serum albumin.

Sphingolipidomic analysis revealed elevated Sph levels in phagocytic cells upon chlamydial infection (Fig. 1a and d). Therefore, we next investigated the spatial distribution of Sph throughout infected M2Φ by employing a functional *ω*-azido-sphingosine (47) combined with 4× ExM. Similarly to Cer (Fig. S3a) and SM (Fig. 5), exogenously added Sph was incorporated into the chlamydial inclusion, as well as the bacterial membranes (Fig. 6a). Likely, endogenous Sph could similarly be transported to *Ctr* membranes.

Since Sph has been shown to have antimicrobial activity against several bacterial and viral species (33, 36, 37, 48), the question arose whether Sph also has anti-chlamydial properties. To answer this question, we analyzed the direct effect of Sph on chlamydial EBs. We used mCherry-expressing purified EBs and treated them with the indicated concentrations of SL for 5 h. Bacterial suspensions were then transferred to freshly plated HeLa 229 cells. Infected HeLa 229 cells were analyzed via flow cytometry 30 h after infection. Treatment with SM or Cer did not substantially alter the infectivity of *Ctr* EBs compared to the untreated control (Fig. 6b). In contrast, treatment with Sph significantly decreased infectivity in a concentration-dependent manner.

Furthermore, we wanted to exclude that the observed effect was due to Sph acting directly on the HeLa 229 cells, e.g., by limiting bacterial uptake itself. To this end, we performed the experimental setup as shown in Fig. 6c. EBs were either treated for 5 h with 50 µM Sph (*t*_0_), or Sph was added simultaneously with the infection (*t*_inf_). Again, the infection rate was analyzed 30 h after infection. We were able to show that the decrease in infectivity was directly linked to the treatment of EBs with Sph and not its effect on HeLa 229 cells, as we observed no difference between untreated chlamydial suspensions and suspensions where we added Sph at t_inf_. On the other hand, there was a significant difference between the Sph added at t_0_ samples and the untreated control, as well as between the Sph added at *t*_inf_ samples. Infectivity decreased as early as 1 h after treatment (Fig. 6d).

We demonstrate that Sph reduces chlamydial infectivity, thus suggesting a potential role for Sph as an anti-chlamydial effector.

## DISCUSSION

SLs are an important class of lipids, both as cellular membrane components and as secondary messengers in various signaling pathways (39). Despite an intact fatty acid and phospholipid biosynthetic machinery (25), the human obligate intracellular pathogen *Ctr* relies on lipid acquisition from the host. SLs, such as Cer and SM, are essential for inclusion stability and intracellular growth of the pathogen (29, 44, 49, 50).

*Chlamydia* preferentially infects epithelial cells, but *in vivo*, *Ctr* is exposed to immune cells at various sites of infection, such as the cervix (51), fallopian tube tissue (52), synovial tissue (53), etc. In recent years, SLs have emerged as key players in host-pathogen interactions (54). Here, we provide the first evidence that host SLs may play a dual role for *Ctr* in infected phagocytes as essential metabolites and immune defense effectors.

Our detailed analysis of SLs that are relevant during the infection of primary macrophages (M1Φ/M2Φ) and PMNs showed that *Ctr* induces cell-type-specific SL signatures in these cells.

We observed particularly drastic changes in M2Φ upon infection. One class of SLs that was drastically increased in M2Φ after infection, but not in other cell types, is Cer. *Ctr* is known to acquire host cell Cer by hijacking SL trafficking pathways (30, 55–57). Analysis of SL distribution in M2Φ using functionalized Cer and SMs showed that both are efficiently taken up into the chlamydial inclusion and are integrated into the bacterial membrane. In epithelial cells, the supposed route for Cer acquisition is uptake via CERT (46), which we found to be recruited to the chlamydial inclusions also in M2Φ. The chlamydial inclusion protein D (IncD) interacts with the amino-terminal pleckstrin homology (PH) domain of CERT, which is also tethered to the ER, thus, presumably forming ER-inclusion MCSs, to enable the transfer of Cer from the ER to the inclusion (45). The inhibition of CERT with HPA-12 was shown to reduce chlamydial inclusion size and to impair progeny formation (44, 45), therefore we suggest that the recruitment of CERT is similarly important for chlamydial development in M2Φ.

To follow SL trafficking in these cells, we made use of TFSMs, which allow tracking of SM metabolization in infected cells (46). Similarly to our previous observations in *Ctr-*infected HeLa cells (46), chlamydial inclusions in M2Φ also harbored TFSMs predominantly in their metabolized form. The metabolization of TFSMs (Fig. 5) indicates the accumulation of SLs that retain the acyl chain: Cer and derivatives (Cer-1-phosphate, hexosyl-, or lactosylCer) but also complex SLs, such as SM (by re-conversion from metabolized Cer) or glycosphingolipids. Since we did not measure an increase in total SMs during infection (Fig. 1), the chlamydial inclusion is likely enriched in Cer and/or hexosyl-, lactosylCer. Of note, during its development, Cer acquisition and its metabolization to SM are critical for chlamydia replication and inclusion stability (29, 44, 49). *Ctr* recruits the host SM synthase to the inclusion (44); however, *Ctr* is also able to synthesize SM in the absence of host SM synthase (58), emphasizing the crucial role of these lipids for *Ctr*.

The mechanism that leads to increased Cer in M2Φ upon *Ctr* infection remains elusive. Cer accumulation can be induced by various stimuli, including exposure to IL-1 or TNF, and follows complex kinetics: from rapid changes within minutes following stimulus exposure to hours (59). Therefore, over the course of the 30 h infection, dynamic changes, including metabolization (e.g., to Sph by acid ceramidases [ASAH1], or SM by SMase [SMPD1]) can affect the outcome of our end-point analysis. Analysis of the expression levels of genes involved in the *de novo* synthesis pathway of Cer in M2Φ did not reveal notable alterations upon *Ctr* infection (Fig. 4), although Cer synthase 5 (CERS5) was upregulated. However, several SL species of the *de novo* pathway, such as dihydrosphingosine (dhSph) and dihydroceramides (dhCer), although not significant, were enriched. Similarly, we did not observe changes in the expression of the lysosomal enzymes *ASAH1* and *SMPD1* which could account for the increased Cer and/or Sph levels upon infection (Fig. 4). Moreover, the compartmentalization of Cer biosynthesis and breakdown (ER, lysosome, plasma membrane [39]) makes it difficult to identify the precise source of Cer increase during *Ctr* infection.

We further analyzed the SL metabolic enzymes to understand the origin of the different SL profiles (Fig. 2). We observed a marked upregulation of *SPHK1* expression in M2Φ, PMNs, but also in FT190 epithelial cells, accompanied by an increase in SPHK enzymatic activity, at both early (2 h, PMN) and late (24 h, M2Φ) infection time points (Fig. 3).

SPHK1 is an oncogenic lipid kinase with pleiotropic effects. It plays a critical role in angiogenesis, signal transduction, apoptosis, or lymphocyte trafficking. SPHK1 has received attention due to its involvement in various malignancies and inflammatory diseases (60).

SPHK1 uses Sph as a substrate to generate S1P. Several studies suggest an important role of SPHK1 and S1P signaling during infection (61–63). The role of S1P as a regulator of host antimicrobial responses is also well documented (64–66). Studies on *C. muridarum*, for example, showed that disruption of S1P signaling (by employing fingolimod, an S1P receptor agonist) delays dissemination of the pathogen from the reproductive to the gastrointestinal tract (67, 68).

An increase in SPHK1 activity was reported under various stress-associated conditions, including, but not limited to LPS-triggered TLR4 signaling (69), hypoxia (70), but also inflammation-induced signaling (71). In both human (THP-1) and mouse macrophages (RAW264.7), SPHK1 was shown to be activated by LPS-triggered TLR4 signaling (69). While chlamydial LPS is not a potent activator of TLR4, heat shock protein 60 (HSP60) from *C. pneumoniae* interacts with TLR4 and myeloid differentiation factor 2 (MD2) in a myeloid differentiation primary response 88 adaptor protein (MyD88)-dependent manner (72). A similar response could be envisioned for *Ctr* to modulate TLR4-mediated pathways.

The most puzzling outcome of our study; however, was the observation that, despite the upregulation of *SPHK1* (and partially *SPHK2*), S1P was not enriched. Instead in M2Φ (with a similar tendency in PMNs), we observed an increase in both Sph and Cer (Fig. 1). S1P is a bioactive molecule, with multifunctional properties under both physiological and pathological contexts. It plays crucial regulatory roles in different immunological processes, including cell survival, innate immunity, and anti-tumor immune response (reviewed by reference 73). It is synthesized by SPHK1 (primarily localized to the cytosol) and SPHK2 (mainly with perinuclear localization) (reviewed by reference 39). The fates of S1P within the host cell include transport to the extracellular domain to act on different types of S1P receptors (74), dephosphorylation into Sph by SGPP phosphatases (in the ER), irreversible cleavage by the SGPL lyase, or degradation of extracellular S1P via lipid phosphate phosphatases at the cell membrane (39). The seeming discrepancy between our lipidome and *SPHK* expression and activity in M2Φ can, at least in part, be explained by the concomitant upregulation of the *SGPL1* lyase, together with the *SGPP1* and *SGPP2* phosphatases (Fig. 2 and 4). In fact, we attribute the increase in Sph, despite elevated SPHK activity, to the massive upregulation of *SGPP2* (Fig. 4). SGPP2, an ER-localized enzyme (75), was shown to be highly upregulated by inflammatory stimuli. Unlike *SGPP1,* the expression of *SGPP2* is reported to be strongly induced by TNF stimulation (76). Since our transcriptomic analysis also revealed increased TNF expression upon infection (log2 fold change = 3.7, adj. *P* < 0.05), we believe that SGPP2 could play a fundamental role in attenuating intracellular S1P signaling during *Ctr* infection of M2Φ.

Therefore, upon *Ctr* encounter, several pathways seem to be simultaneously induced. In the “tug of war” between host and pathogen, pathogen-associated molecular patterns (PAMPs) are likely to induce SPHKs, as a general and rather unspecific host response (61–63, 77). Several signals which upregulate SPHK, however, are also likely to regulate SGPP and SGPL and, consequently, opposing pathways.

A striking observation in this study was that direct incubation with Sph reduces the infectivity of *Ctr* EBs. This is in agreement with several reports attributing antimicrobial properties to Sph against intracellular *N. gonorrhoeae* (36)***,*** *Propionibacterium acnes, Micrococcus luteus* (78), *Fusobacterium nucleatum,* or *Streptococcus mitis* (33)*,* among others.

The M1Φ, owing to its extremely microbicidal phenotype, completely abrogates *Ctr* growth, likely shortly after initial contact with the pathogen. Therefore, all subsequent responses to the *Ctr* infection are dampened, which results in minimum alterations of both lipidome and host gene expression. By contrast, both M2Φ and epithelial cells are permissive niches for the pathogen. However, the dynamics of the infection are different. While epithelial infection is very efficient and *Ctr* establishes a stable replicative niche (~60% infection rate), only a subpopulation of M2Φ supports *Ctr* infection. M2Φ allows inclusion formation, *Ctr* replication, and production of infectious progeny; however, infection rates are very low (~8%) (Fig. S1). As the results of both lipidome and gene expression experiments represent the outcome of the bulk analysis of a heterogeneous M2Φ population, we propose that the global response that we observe might in fact look different at the single-cell level. We envision that the uninfected or bystander cells, which do not carry inclusions, result in an abortive *Ctr* infection. The enriched Sph levels we measure could originate from these cells, which represent the majority of the analyzed population. Our model proposes that the increase in SPHK transcripts and activity is a hallmark of a generalized host response against PAMPs of both bacterial and viral origin, rather than a specific response to *Ctr* infection. We cannot, however, exclude that SPHK1 plays a role beyond its kinase function in this context, such as endocytic membrane trafficking (79, 80) or acetyltransferase activity in the presence of Sph as substrate (81). While the role of *SPHK* upregulation upon *Ctr* infection remains elusive, it is tempting to speculate that, due to the heterogeneity of *Ctr* infection in M2Φ, different signatures, both metabolic and transcriptional, can be attributed to the different cells present within the analyzed population: infected, uninfected, and bystanders. As macrophages are known to exhibit remarkable phenotypic plasticity (19), *Ctr* might be able to exploit the more permissive cells within the population for growth and development, while the majority of the population restricts the pathogen (e.g., by maintaining a high pool of Sph via the phosphatase activity of SGPP1/2). A systematic examination of the role of each of the described pathways at the single-cell level could reveal the role of lipidome remodeling, in both professional phagocytes and epithelial cells, in complex tissues where several cell types are encountered by the pathogen.

## MATERIALS AND METHODS

### Cell lines and bacteria

HeLa 229 (ATCC CCL-2.1) and hTERT FT 190 (ATCC CRL-3444) cells were cultured in RPMI 1640 Medium with GlutaMAX supplement (Gibco, #61870-044) in the presence of 10% (vol/vol) heat-inactivated (30 min, 56°C) fetal bovine serum (FBS, Sigma Aldrich, #A5256701) (standard medium).

For cell culture maintenance, standard tissue culture procedures were used. Cells were cultured in a humidified atmosphere with 5% CO_2_ (vol/vol) at 37°C. For this study, *Chlamydia trachomatis* serovar L2/434/Bu (ATCC VR-902B) and L2/434/Bu (ATCC VR-902B) transformed with mCherry were used. HeLa 229 cells and chlamydial EBs were routinely tested for *Mycoplasma* contamination by PCR. All cell lines were authenticated by ATCC, therefore no further validation was performed in our laboratory.

### Purification of chlamydial EBs

Preparation of clear lysates was performed as published before (82). In brief, HeLa 229 cells were used to expand *Chlamydia trachomatis* (*Ctr*) L2 for 48 h. Using glass beads (2.85–3.45 mm, Roth, #A557.1), cells were disrupted and mechanically lysed. Bacteria were collected by centrifugation for 10 min at 755 × *g* at 4°C and supernatants were then centrifuged for 30 min at 40,000 × *g* at 4°C. The pellet was washed once with 1× sucrose-phosphate-glutamic acid (SPG) buffer (7.5% sucrose [Roth, #4621.2], 0.05% KH_2_PO_4_ [Roth, #3904.1], 0.12% Na_2_HPO_4_ [Roth, #P030.2], 0.072% L-glutamine [Gibco, #25030081]). The pellet was resuspended in 1× SPG buffer and passed through 20 G (B. Braun, #612-0141) and 18 G (B. Braun, #612-0147) hollow needles. Bacteria were stored at −80°C until needed. For RNAseq experiments, *Ctr* L2 were subsequently purified by Gastrografin (Bayer Vital GmbH Germany, #86971488) density gradient purification, using 20% (vol/vol) and 50% (vol/vol) Gastrografin in Hanks’ buffered saline (HBSS) (Thermo Scientific, #14025-100). Following centrifugation (1 h at 60,000 × *g* at 4°C), *Ctr* pellets were re-suspended in SPG buffer, passed through 20 G and 18 G hollow needles, and stored at −80°C. In parallel, uninfected HeLa 229 were handled similarly to obtain *Ctr-*free Mock samples.

### Cloning of *C. trachomatis* L2 mCherry

The *aad*A gene was introduced into pBOMB4-mCherry (83) to allow clone selection by spectinomycin resistance. The *aad*A gene was amplified by PCR from plasmid pSUmC4.0 (84) (for primer sequences, see Table S1). The amplified *aad*A gene and plasmid pBOMB4-mCherry were digested with restriction enzymes KpnI (Thermo Scientific, # FD0524) and SalI (Thermo Scientific, # FD0644) and transformed into *E. coli* DH5α (Thermo Scientific, #EC0112) after ligation. Positive clones were electroporated into *E. coli* JM110 and subsequently transformed into *Ctr* L2 according to a previously published method with spectinomycin selection (84).

### Isolation of human neutrophils (PMNs)

Isolation of PMNs was performed as published before (9). In brief, venous blood was collected from healthy donors in lithium heparin tubes (Sarstedt, #02.1065). Blood cells were separated using a Ficoll Paque Plus (Cytiva, # GE17-1440-03) gradient and centrifugation at 425 × *g* for 30 min at RT without brakes. The plasma layer was collected, centrifuged for 10 min at 755 × *g* at RT, sterile filtered using a syringe filter with 0.2 µm pore size (Sarstedt, #83.1826.001) and diluted to 10% (vol/vol) with 1× Dulbecco’s phosphate-buffered saline (DPBS; Gibco, # 14190169). All cell culture plates and tubes used for culturing the cells were coated with 10% autologous plasma for 30 min at RT, then washed twice with 1× DPBS. The layer containing peripheral blood mononuclear cells and the Ficoll layer were aspirated. A 1% polyvinyl alcohol solution (0.85% sodium chloride [Roth, #P029.1], 1% polyvinyl alcohol [Sigma-Aldrich, #341584-25G]) was used to sediment erythrocytes for 45 min at RT. The supernatant was centrifuged for 5 min at 188 × *g* at RT, and erythrocytes were lysed with sterile water. A 5× DPBS was used to restore osmolarity. Neutrophils were collected by centrifugation for 5 min at 188 × *g* at RT. PMNs were resuspended in RPMI 1640 medium with GlutaMAX supplement at a density of 10^6^ cells/mL and seeded in the designated cell culture plate or tube. Cells were allowed to rest 30 min prior to other experimental procedures. PMNs were cultured in a humidified atmosphere with 5% CO_2_ (vol/vol) at 37°C.

### Isolation of human monocytes and macrophage differentiation

Primary human macrophages were derived from PBMCs, isolated from leukoreduction system cones using the SepMate-50 system (StemCell Technologies, #85450) and Ficoll-Paque Plus (Cytiva, # GE17-1440-03) gradient according to manufacturer’s instructions.

Monocytes were purified from PBMCs using the EasySep CD14+ system (StemCell Technologies, #17858), according to the manufacturer’s instructions, and subsequently cultivated in a *standard medium*. Culture medium was supplemented with 50 ng/mL recombinant human macrophage colony-stimulating factor (M-CSF) (StemCell Technologies, #78057), for M2Φ, or 25 ng/mL granulocyte-macrophage colony-stimulating factor (GM-CSF) (StemCell Technologies, #78190), for M1Φ. After 7 days, macrophage polarization was induced by a 48 h treatment with either 100 ng/mL IL-4 (StemCell Technologies, #78045.1) for “M2Φ” polarization or 100 ng/mL LPS (Sigma Aldrich, # L6529) and 50 ng/mL IFNγ (PeproTech, #AF-300-02) for “M1Φ” polarization. Cells were cultured in a humidified atmosphere with 5% CO_2_ (vol/vol) at 37°C and used for infection experiments on day 9 of differentiation.

### Infection with *Chlamydia trachomatis*

Different multiplicities of infection (MOI) were used. HeLa 229 cells, FT190 cells, M2Φ, and M1Φ, and were infected at an MOI of 1, and PMNs were infected at an MOI of 10. M2Φ/M1Φ and FT190 cells were centrifuged for 10 min at 188 × *g* at RT after infection. For M2Φ/M1Φ and FT190, infection was allowed to proceed for 2 h, after which cells were washed 1× with DPBS, and fresh standard medium was added.

### Quantification of sphingolipids by liquid chromatography tandem‐mass spectrometry

A total of 10^6^ cells were left uninfected or were infected *with Ctr* serovar L2 for 24 h (PMNs) or 30 h (M2Φ, M1Φ, FT190). Cells were resuspended in 500 µL methanol (LC-MS CHROMASOLV, Fluka analytical #34966-1L) and subjected to sphingolipid extraction. To this end, 1 mL methanol/chloroform (1:1, vol:vol) was added that contained the internal standards d_7_-dihydrosphingosine (d_7_-dhSph), d_7_-sphingosine (d_7_-Sph), d_7_-sphingosine 1-phosphate (d_7_-S1P), 17:0 ceramide (d18:1/17:0), d_31_-16:0 sphingomyelin (d18:1/16:0-d_31_), 17:0 glucosyl(β) ceramide (d18:1/17:0), and 17:0 lactosyl(β) ceramide (d18:1/17:0) (all from Avanti Polar Lipids, Alabaster, AL, USA) (85). Final extracts were subjected to liquid chromatography tandem‐mass spectrometry (LC-MS/MS) sphingolipid quantification applying the multiple reaction monitoring approach. Chromatographic separation was achieved on a 1290 Infinity II HPLC (Agilent Technologies, Waldbronn, Germany) equipped with a Poroshell 120 EC-C8 column (3.0 × 150 mm, 2.7 µm; Agilent Technologies) guarded by a pre-column (3.0 × 5 mm, 2.7 µm) of identical material. MS/MS analyses were carried out using a 6495C triple-quadrupole mass spectrometer (Agilent Technologies) operating in the positive electrospray ionization mode (ESI+). Chromatographic conditions and settings of the ESI source and MS/MS detector have been published elsewhere (86). The mass transitions used for the analysis of sphingolipid subspecies are given in Table S2. Peak areas of Cer, dhCer, SM, dhSM, HexCer, and LacCer subspecies, as determined with MassHunter software (Agilent Technologies), were normalized to those of their internal standards followed by external calibration. DhSph, Sph, and S1P were directly quantified via their deuterated internal standards. Quantification was performed with MassHunter Software (Agilent Technologies).

### Sphingosine kinase activity assay

A total of 10^6^ PMNs, 5 × 10^4^ M2Φ, and 1 × 10^5^ FT190 cells, respectively, were used per condition and left uninfected or infected with *Ctr* L2 for the designated time points. Sphingosine kinase activity was measured using the Sphingosine Kinase Activity Assay (Echelon Biosciences, #K-3500-EAKIT-EC). Cells were lysed by 3 × 10 min freezing (dry ice) and thawing (37°C) cycles. The assay was performed according to the manufacturer’s protocol. Luminescence was measured with the Tecan Infinite M200 microplate reader using the Tecan i-control software (version 1.12.4.0).

### RT-qPCR

A total of 30 × 10^6^ PMNs, 10^6^ M2Φ, or 5 × 10^5^ FT190 were used per condition and left uninfected or infected with *Ctr* L2 for 2, 24, or 30 h. RNA was isolated using the RNeasy Mini Kit (Qiagen, #74104) or TRIzol Reagent (Invitrogen, #15596026) in the case of PMNs, according to the manufacturer’s protocols. If RNA was isolated using the Qiagen kit, the DNA was removed using the RNase-Free DNase Set (Qiagen, # 79254). In the case of PMNs, DNA was removed using the TURBO DNA-*free* Kit (Invitrogen, # AM1907). cDNA was generated using the Revert Aid First Strand cDNA Synthesis Kit (Thermo Scientific, #K1622). RT-qPCR was performed according to the StepOne software (version 2.3) using the GreenMasterMix (2X) High ROX (Gennaxon, #M3052.0500) and the StepOnePlus system (Life Technologies). Data was reanalyzed using the Design & Analysis Software 2.7.0 (Thermo Scientific). Data analysis was done according to the 2^(−ΔΔCt)^ method (87) using Excel (Microsoft). *UBC* (PMN) or *YWHAZ* (M2Φ, M1Φ, FT190) was used as endogenous control. The used primers are shown in Table S3.

### RNA sequencing of M2Φ infected with *C. trachomatis* L2

A total of 10^6^ M2Φ were infected with *Ctr* serovar L2 or Mock-treated for 30 h as described. For RNA isolation, cells were washed once with DPBS and immediately 100 µL fresh standard medium and 500 µL RNAprotect Cell Reagent (Qiagen, #76526) were added. Detached cells were collected by centrifugation for 5 min at 10,000 × *g* at 4°C and RNA isolation was performed using the RNeasy Mini Kit (Qiagen, #74104) and according to manufacturer’s instructions. RLT Buffer (kit) was supplemented with beta-mercaptoethanol. Lysis Matrix B tubes (MP Biomedicals, #116911100) were used to ensure complete sample disruption. DNA was removed using the TURBO DNA-*free* Kit (Invitrogen, # AM1907).

Ribosomal RNA depletion was done using RiboCop for Human/Mouse/Rat V2 (Cat. No. 144) and RiboCop META (Cat. No. 125) rRNA depletion kit (Lexogen) according to the manufacturer’s instructions (½ volume). The ribo-depleted RNA samples were first fragmented using ultrasound (4 pulses of 30 s at 4°C). Then, an oligonucleotide adapter was ligated to the 3′ end of the RNA molecules. First-strand cDNA synthesis was performed using M-MLV reverse transcriptase with the 3′ adapter as a primer. After purification, the 5′ Illumina TruSeq sequencing adapter was ligated to the 3′ end of the antisense cDNA. The resulting cDNA was PCR-amplified using a high-fidelity DNA polymerase, and the barcoded TruSeq libraries were pooled in approximately equimolar amounts. Sequencing of pooled libraries spiked with PhiX control library, was performed at 50 million reads per sample in single-ended mode with 100 cycles on the NextSeq 2000 platform (Illumina). Demultiplexed FASTQ files were generated with bcl-convert v4.2.4 (Illumina). Raw sequencing reads were quality- and adapter-trimmed via Cutadapt (88) v2.5 using a cutoff Phred score of 20 in NextSeq mode, and reads without any remaining bases were discarded (parameters: --nextseq-trim=20 -m 1 -a AGATCGGAAGAGCACACGTCTGAACTCCAGTCAC). Processed reads were unambiguously assigned to either human or *Chlamydia trachomatis* via FastQ Screen (89) v0.15.3 with parameters --aligner bowtie2 --tag for read tagging and --filter “03” and “30” to generate split FASTQ read files for *Chlamydia* and human, respectively. The parameter --subset 0 was applied to process all reads instead of only a subset. For this and subsequent analyses, NCBI RefSeq assemblies GCF_000001405.40/GRCh38.p14 (primary assembly and mitochondrion) for humans as well as GCF_000364765.1 for *Chlamydia trachomatis* L2 were used. We mapped the human-assigned reads to the reference genome using STAR (90) v2.7.2b with default parameters except for including transcript annotations from RefSeq annotation version RS_2023_03 for GRCh38.p14. This annotation was also used to generate read counts on the exon level summarized for each gene via featureCounts v1.6.4 from the Subread package (91). Multi-mapping and multi-overlapping reads were counted stranded with a fractional count for each alignment and overlapping feature (parameters: -s 1 -t exon -M -O --fraction). The count output was utilized to identify differentially expressed genes using edgeR (92) v4.0.16.

Differential expression data were further analyzed with R Studio (93, 94). Gene annotations were accessed from Gene Ontology (40, 42) for the categories sphingolipid biosynthetic processes (GO:0030148), sphingolipid catabolic processes (GO:0030149), sphingolipid mediated signaling (GO:0090520), sphingolipid transfer activity (GO:0120016), and sphingolipid metabolic processes (GO:0006665). Differentially expressed genes (*Ctr* vs Mock) were compared to genes that were retrieved for each category and analyzed for significant regulation (adj. *P* < 0.05, log2 fold change > 0.5 or < −0.5) upon *Ctr* infection. Graphs (Fig. 4a and b) were generated with ggplot2 (95).

### BSA complexing of sphingolipids

The following protocol was adapted from references 96 and 97. In brief, 1 mM stock solutions of D-*erythro* sphingosine (Santa Cruz, # sc-3546A), sphingomyelin (Avanti Polar Lipids, #860061P), and C_16_ ceramide (Avanti Polar Lipids, #860516P) were prepared. 6.8 mg (0.1 mM) bovine serum albumin (fatty-acid free, low endotoxin, Sigma Aldrich, # A8806) was dissolved in 1 mL 100 mM NaH_2_PO_4_ (VWR, # 28014.291)/Na_2_HPO_4_ (Roth, # P030.2), pH 7.4. The respective amount of lipid was dissolved in 20 µL ethanol (Puriss p.a. absolute 99.8% (GC), Sigma Aldrich, # 32,221-M). While vortexing, the lipid solution was injected into the bovine serum albumin (BSA) solution. For the BSA control solution, 20 µL ethanol was injected into the BSA solution. After aliquoting, stock solutions were kept at −20°C until use.

### Sphingolipid treatment of *Chlamydia* EBs

Ctr L2 mCherry EBs in an amount considered as MOI 1 for the number later used for HeLa 229 cells were left untreated or were treated with the designated concentration of BSA control, SM, Cer, or Sph in standard medium for 5 h or the designated time points. Bacteria were maintained in a humidified atmosphere with 5% CO_2_ (vol/vol) at 35°C. The bacterial suspension was transferred to freshly plated HeLa 229 cells and *Ctr* was propagated for 30 h. Cells were detached using TrypLE Express (Gibco, # 12604039) and resuspended in 300 µL 1× DPBS. The presence of mCherry positive cells was analyzed using the Attune NxT acoustic focusing cytometer (Thermo Fisher Scientific): YL2 channel, excitation 561 nm/emission 620 ± 15 nm band-pass. A total of ~10^4^ intact cells were analyzed, and mCherry-positive events were gated on the single cell population (FSC-H/FSC-A).

### Infection rate analysis

M2Φ and FT190 cells were infected with *Ctr* L2 mCherry at an estimated MOI of 1. In this experiment, ~8 × 10^6^ monocytes were seeded and differentiated and polarized to M2Φ, in 100 mm Nunc Dishes with UpCell Surface (Thermo Fisher, #174902), to allow subsequent detachment of the cells without enzymatic treatment or scraping. Infection was allowed to proceed for 2 h, after which cells were washed 1× with DPBS and fresh standard medium was added. At 30 h p.i., infected and uninfected cells were detached according to the manufacturer’s instructions (for M2Φ) or using TrypLE (for FT190). The presence of mCherry positive cells was analyzed using the Attune NxT acoustic focusing cytometer (Thermo Fisher Scientific): YL2 channel, excitation 561 nm/emission 620 ± 15 nm band-pass. A total of ~10^4^ intact cells were analyzed, and mCherry-positive events were gated on the single-cell population (FSC-H/FSC-A).

### Antibody staining for confocal laser scanning microscopy

All used lipids, antibodies, and dyes are listed in Table S4. A total of 10^5^ M2Φ were used per condition and were infected with *Ctr* L2 for 30 h at MOI 1. For confocal laser scanning microscopy, cells were fixed with a 4% paraformaldehyde solution (PFA, Morphisto, #11762.01000) for 15 min. Cells were permeabilized with 0.02% Triton X-100 (Roth, # 3051.4) in 1× DPBS for 15 min. Cells were incubated for 1 h in a blocking buffer (1× DBPS containing 2% FBS). Cells were stained against chlamydial HSP60 and human CERT for 1 h at RT and afterward co-stained for 1 h with fluorophore-coupled secondary antibodies. DNA was stained using DAPI. After each staining step, three washes with 1× DPBS were performed. Before embedding, the coverslips were shortly dipped in ddH_2_O for desalting. Coverslips were embedded in Mowiol (24 g glycerol [Roth, # 3783.2], 9.6 g Mowiol 4-88 [Roth, # 0713.2], 48 mL 0.2 M Tris-HCl pH 8.5 [Sigma # T1503], 24 mL Millipore H_2_O). The TCS SP5 confocal laser scanning microscope (Leica, Wetzlar, Germany) was used for imaging.

### 4× expansion microscopy of functionalized sphingolipids

All used lipids, antibodies, and dyes are listed in Table S4. Lipid staining and expansion were performed as published before (43, 46, 98). In brief, 10^5^ M2Φ were used per condition and were infected with *Ctr* L2 for 30 h at MOI 1. If Cer or Sph were used for analysis, cells were incubated with 10 µM SL 1 h prior to fixation. If SM was analyzed, 10 µM SM was added 3 h post-infection. Samples were fixed using 4% PFA/0.2% glutaraldehyde (GA; Sigma Aldrich, #G5882) in DPBS and washed three times. After permeabilization with 0.02% Triton X-100 and washing thrice, functionalized lipids were stained. SM was first stained with 4 µM BODIPY-FL-DBCO in Hanks’ buffered saline (HBSS; Gibco, # 14025-100) for 1 h at 37°C, followed by 20 µM AlexaFluor 546 azide in 50 µM CuSO_4_/2.5 mM sodium ascorbate [Sigma Aldrich, #A4034)/250 µM tris(3-hydroxypropyltriazolylmethyl)amine (THPTA; Sigma Aldrich, #762342] in DPBS for 1 h at 37°C. Cer and Sph were stained with 10 µM AlexaFluor 488-DBCO in HBSS for 1 h at 37°C. After each lipid staining step, cells were washed five times with 1× DPBS. After lipid staining, cells were incubated for 1 h in blocking buffer and then stained against chlamydial HSP60 for 1 h at RT and co-stained for 1 h with fluorophore-coupled secondary antibodies. Samples were fixed using 4% PFA/0.2% GA. The cells were placed in 56 µL monomer solution (8.625% sodium acrylate [Sigma Aldrich, # 408220]/2.5% acrylamide [Sigma Aldrich, # A9926]/0.15% N,N′-methylene bisacrylamide [Sigma Aldrich, # 146072], 2 M NaCl [Sigma Aldrich, # S5886] in DPBS) that was freshly supplemented with 0.2% (wt/vol) ammonium persulfate (Sigma Aldrich, #A3678) and 2% (vol/vol) tetramethyl ethylene diamine (Sigma Aldrich, #T9281-100ML) after 75 min of polymerization proteinase K digestion (8 U/mL (Sigma Aldrich, #P4860) in 50 mM Tris (pH 8.0, 1 mM EDTA [Sigma Aldrich, # ED2P]/0.5% Triton X-100/0.8 M guanidine HCl [Sigma Aldrich, #50933]) was carried out for 1 h. Expansion of gels was carried out in ddH_2_O overnight, including at least two water exchanges. Gels were imaged in a Lab-TekTM II chamber (VWR, # 734-2055) that was coated with 0.01% poly-L-lysine solution (Sigma Aldrich, #A-005-C). The TCS SP5 confocal laser scanning microscope was used for imaging.

### FRET acceptor bleaching

The built-in FRET AB wizard of the Leica TCS SP5 microscope was used to perform FRET acceptor bleaching. Images in both donor and acceptor channels were recorded, followed by the determination of the region of interest to allow selected acceptor fluorophore bleaching by recording 20 frames with high laser intensity. Again, both channels were recorded. FRET efficiency was calculated by comparing donor and acceptor signal pre- and post-bleaching via the following formula:


$$FRET_{eff}.=\left[\frac{\left(donor_{post}-donor_{pre}\right)}{donor_{post}}\;\right]\times \;100.$$

### Statistical methods

Statistical analysis was carried out for at least three independent biological replicates, using GraphPad Prism Software (Version 10.2.3). Sample size and used statistical tests are indicated in each figure legend.

## Data Availability

Data have been deposited at the European Genome-phenome Archive (EGA), which is hosted by the EBI and the CRG, under accession number EGAS50000000960. Further information about EGA can be found at https://ega-archive.org and "The European Genome-phenome Archive of human data consented for biomedical research."

## SUPPLEMENTAL MATERIAL

Data Set S1 mbio.03981-24-s0001.xlsx Overview of sphingolipid-associated genes with significantly changed expression during *Chlamydia* infection.

Supplemental Material mbio.03981-24-s0002.docx Figures S1–S3, Tables S1–S4, and legend to Data Set S1.

ASM does not own the copyrights to Supplemental Material that may be linked to, or accessed through, an article. The authors have granted ASM a non-exclusive, world-wide license to publish the Supplemental Material files. Please contact the corresponding author directly for reuse.
