# Supplementary material for: Chlamydia trachomatis exploits sphingolipid metabolic pathways during infection of phagocytes
Source: mBio. 2025 Apr 18;16(5):e03981-24. doi: 10.1128/mbio.03981-24 (PMC12077188; doi:10.1128/mbio.03981-24)
Supplement: Supplemental Material — Figures S1–S3, Tables S1–S4, and legend to Data Set S1. [file mbio.03981-24-s0002.docx]

**SUPPLEMENTAL MATERIAL for**

***Chlamydia trachomatis* exploits sphingolipid metabolic pathways during infection of phagocytes**

Adriana Moldovan^a+^, Fabienne Wagner^a+^, Fabian Schumacher^b^, Dominik Wigger^b*1^, David Komla Kessie^a^, Marcel Rühling^a^, Kathrin Stelzner^a^, Regina Tschertok^a^, Louise Kersting^c*2^, Julian Fink^c^, Jürgen Seibel^c^, Burkhard Kleuser^b^, Thomas Rudel^a^**^#^**

A.M.^+^ and ^+^F.W. and contributed equally to this work. Author order was determined based on a random draw.

Supplemental Material includes:

Supplemental Figures S1-S3

Supplemental Tables S1-S4

Supplemental References

Legend to Supplemental Data Set 1 (.xlsx file)

# **Supplemental Figures**

**
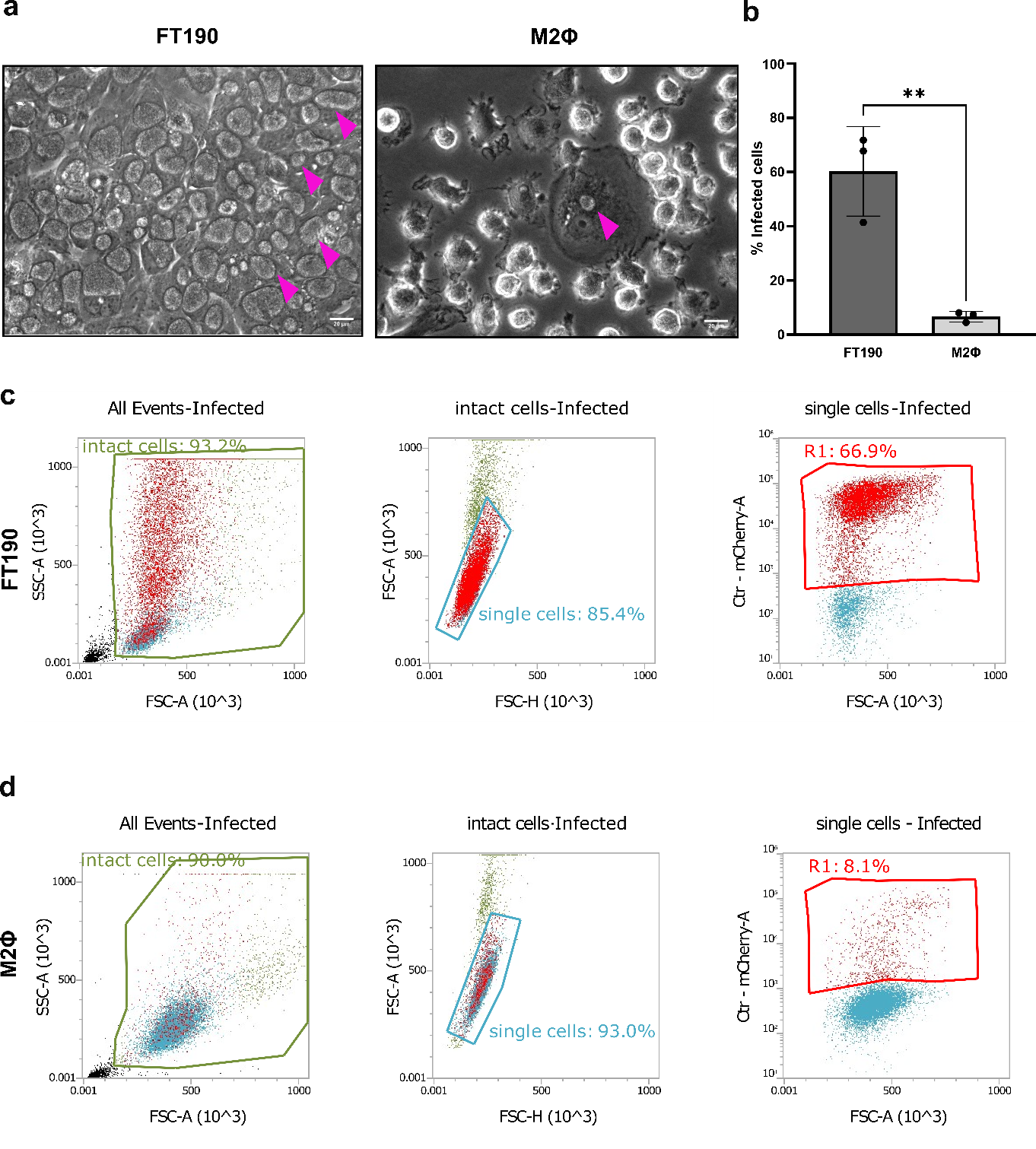
**

**Figure S1 *C. trachomatis* infection rate**

**(a)** Phase-contrast image of FT190 epithelial cells or M2Φ infected with *Ctr* (30 h p.i.). Arrows indicate presence of *Ctr*-containing inclusions. Scale bar 20 µm. **(b)** Flow-cytometry analysis of FT190 or M2Φ cells infected with *Ctr* L2 mCherry (30h p.i) at an estimated MOI=1. Bar graphs represent the percent (%) of mCherry-positive events from the total recorded single cells. Data are shown as mean ± SD, from independent biological replicates (n=3). Unpaired, two-tailed t-test was used for analysis. (** p < 0.01). (**c** and **d**) Gating strategy used for determining infection rates shown in (**c**). ~10^4^ intact cells were analyzed, and mCherry-positive events were gated on the single cell population (FSC-H/FSC-A). Representative plots are shown. (*M2Φ: M2-like primary human macrophages*).

**
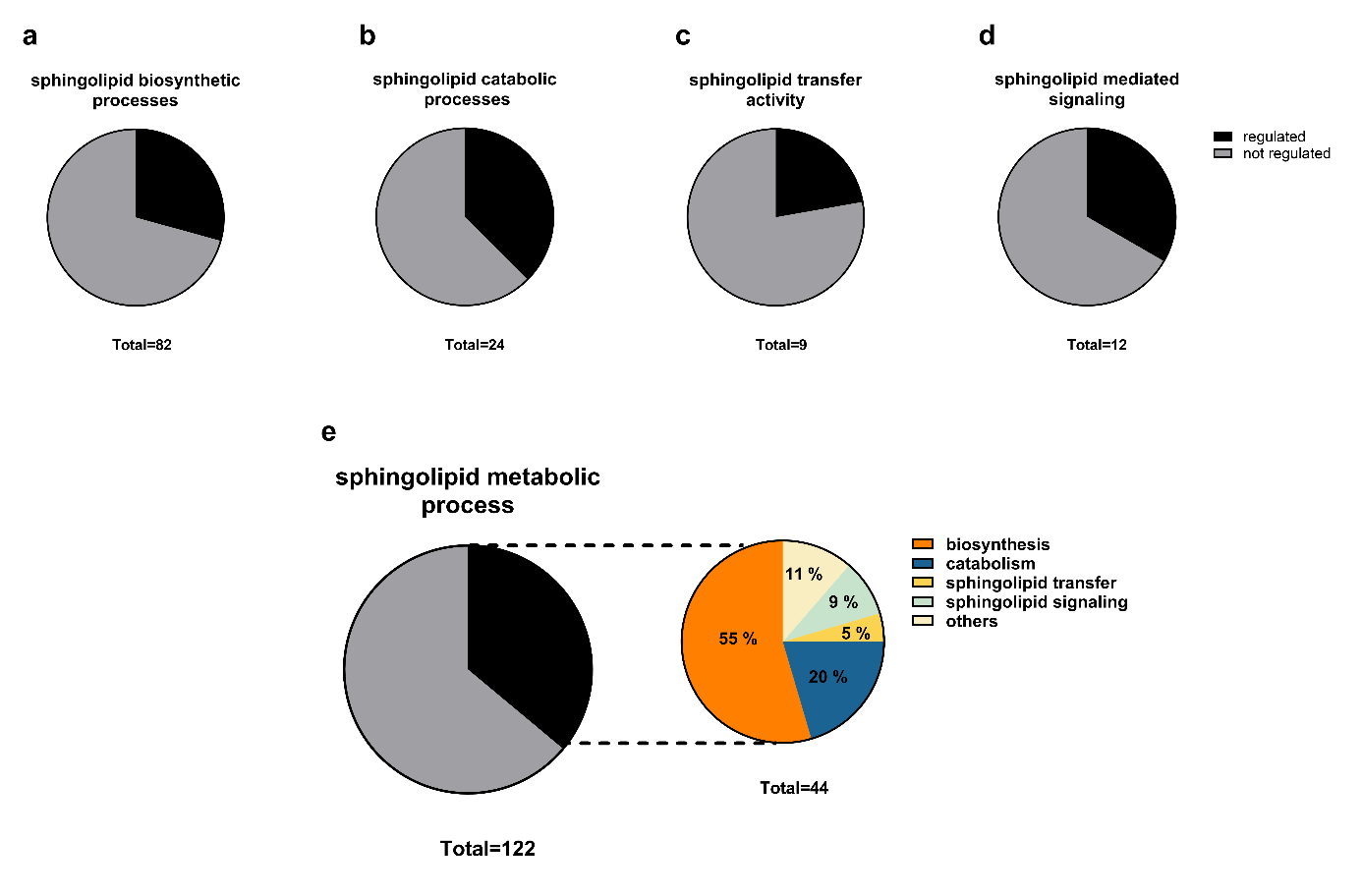
**

**Figure S2 Categorization of sphingolipid-associated genes regulated upon *C. trachomatis* infection**

Genes annotated in the categories **(a)** s*phingolipid biosynthetic processes* (GO:0030148), **(b)** *sphingolipid catabolic processes* (GO:0030149), **(c)** *sphingolipid transfer activity* (GO:0120016) **(d)** *sphingolipid mediated signaling* (GO:0090520) and **(e)** *sphingolipid metabolic processes* (GO:0006665) were accessed from Gene Ontology [1, 2]. Pie charts illustrate the proportion of genes in each category that were detected as significantly regulated upon *Ctr* infection in M2Φ macrophages (30 h p.i.) (adj. p< 0.05 and log2 fold change >0.5 or <-0.5). The total number of detected genes assigned to the respective category is indicated below the graphs. The inlet in **(e)** illustrates the distribution of 44 sphingolipid-related genes significantly regulated during *Ctr* infection among the categories.

**
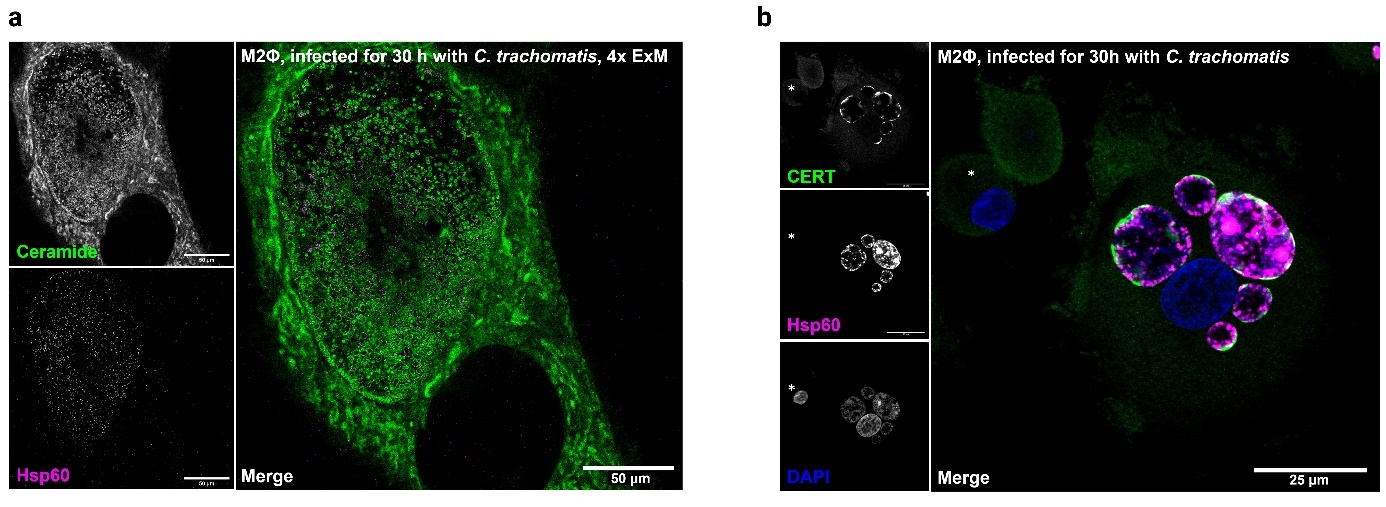
**

**Figure S3: Ceramide is incorporated into the chlamydial membrane**
**(a)** Confocal fluorescence image of a 4x expanded **M2Φ** infected with *Ctr* (30 h p.i.). *α*-amino-*ω*-azido-C_6_-ceramide was added 1 h prior to fixation. Cer was stained with DBCO-AF488 via click chemistry (green), *Ctr* were stained against the chlamydial heat shock protein 60 (Hsp60, magenta). Scale bar 50 µm. **(b)** Confocal fluorescence images of an **M2Φ** infected with *Ctr* (30 h p.i.). Ceramide transfer protein (CERT) was stained (magenta), *Ctr* were stained against Hsp60 (green) and DNA was stained with DAPI (blue). Scale bar 25 µm. The asterisk marks a not infected cell for comparison.

# **Supplemental Tables**

Table S1: Primers used for cloning of *C. trachomatis* L2 mCherry

| **Name** | **Sequence 5‘-3‘** | **Source** |
| --- | --- | --- |
| aadA+KpnI_fwd | aaGGTACCtcatcatgcctcctctagaccag | **This study** |
| aadA+SalI_rev | aaagtcgacTTACCAATGCTTAATCAGTGAGGC | **This study** |

Table S2. LC-MS/MS parameters for sphingolipid quantification.

| **Sphingolipid** | **Precursor ion (*m/z*)** | **Product ion (*m/z*) a** | **Retention time (min)** | **Internal Standards** |
| --- | --- | --- | --- | --- |
| d_7_-Sph | 307.3 [M+H]+ | **289.3 (8)** / 259.3 (20) | 5.5 | - |
| d_7_-dhSph | 309.4 [M+H]+ | **291.3 (12)** / 261.3 (24) | 5.8 | - |
| d_7_-S1P | 387.3 [M+H]+ | **271.3 (20)** / 82.1 (36) | 6.8 | - |
| Sph | 300.3 [M+H]+ | **282.3 (8)** / 252.3 (16) | 5.5 | d_7_-Sph |
| dhSph | 302.3 [M+H]+ | **284.3 (12)** / 254.3 (20) | 5.8 | d_7_-dhSph |
| S1P | 380.3 [M+H]+ | **264.3 (20)** / 82.1 (32) | 6.8 | d_7_-S1P |
| 16:0 dhCer | 540.5 [M+H]+ | **522.6 (20)** / 284.3 (28) | 14.2 | 17:0 Cer |
| 18:0 dhCer | 568.5 [M+H]+ | **550.5 (20)** / 284.3 (28) | 16.3 | 17:0 Cer |
| 20:0 dhCer | 596.6 [M+H]+ | **578.6 (22)** / 284.3 (32) | 18.9 | 17:0 Cer |
| 22:0 dhCer | 624.6 [M+H]+ | **606.6 (22)** / 284.3 (32) | 22.1 | 17:0 Cer |
| 24:0 dhCer | 652.7 [M+H]+ | **634.6 (24)** / 284.3 (36) | 25.7 | 17:0 Cer |
| 24:1 dhCer | 650.7 [M+H]+ | **632.7 (24)** / 284.3 (36) | 22.6 | 17:0 Cer |
| 17:0 Cer | 534.5 [M-H2O+H]+ | **264.3 (24)** / 282.3 (28) | 14.6 | - |
| 16:0 Cer | 520.5 [M-H2O+H]+ | **264.3 (24)** / 282.3 (24) | 13.7 | 17:0 Cer |
| 18:0 Cer | 548.5 [M-H2O+H]+ | **264.2 (24)** / 282.3 (28) | 15.6 | 17:0 Cer |
| 20:0 Cer | 576.6 [M-H2O+H]+ | **264.3 (32)** / 282.3 (28) | 18.0 | 17:0 Cer |
| 22:0 Cer | 604.6 [M-H2O+H]+ | **264.3 (34)** / 282.3 (30) | 21.0 | 17:0 Cer |
| 24:0 Cer | 632.6 [M-H2O+H]+ | **264.3 (36)** / 282.3 (28) | 24.5 | 17:0 Cer |
| 24:1 Cer | 630.6 [M-H2O+H]+ | **264.3 (36)** / 282.3 (32) | 21.2 | 17:0 Cer |
| 16:0 dhSM | 705.6 [M+H]+ | **184.0 (8)** / 86.1 (76) | 13.5 | d_31_-16:0 SM |
| 18:0 dhSM | 733.6 [M+H]+ | **184.0 (28)** / 86.1 (76) | 15.6 | d_31_-16:0 SM |
| 20:0 dhSM | 761.6 [M+H]+ | **184.0 (28)** / 86.1 (78) | 18.0 | d_31_-16:0 SM |
| 22:0 dhSM | 789.7 [M+H]+ | **184.0 (28)** / 86.1 (78) | 20.9 | d_31_-16:0 SM |
| 24:0 dhSM | 817.7 [M+H]+ | **184.0 (28)** / 86.1 (80) | 24.5 | d_31_-16:0 SM |
| 24:1 dhSM | 815.7 [M+H]+ | 184.0 (8) **/ 86.1 (80)** | 20.9 | d_31_-16:0 SM |
| d_31_-16:0 SM | 734.6 [M+H]+ | **184.0 (28)** / 86.1 (76) | 12.7 | - |
| 16:0 SM | 703.6 [M+H]+ | **184.0 (8)** / 86.1 (76) | 12.8 | d_31_-16:0 SM |
| 18:0 SM | 731.6 [M+H]+ | **184.0 (28)** / 86.1 (76) | 14.7 | d_31_-16:0 SM |
| 20:0 SM | 759.6 [M+H]+ | **184.0 (28)** / 86.1 (78) | 17.0 | d_31_-16:0 SM |
| 22:0 SM | 787.7 [M+H]+ | **184.0 (28)** / 86.1 (78) | 19.4 | d_31_-16:0 SM |
| 24:0 SM | 815.7 [M+H]+ | **184.0 (28)** / 86.1 (80) | 22.7 | d_31_-16:0 SM |
| 24:1 SM | 813.7 [M+H]+ | 184.0 (8) / **86.1 (80)** | 19.5 | d_31_-16:0 SM |
| 17:0 Glucosyl-Cer | 714.6 [M+H]+ | **264.2 (44)** / 696.6 (12) | 13.2 | - |
| 16:0 Hexosyl-Cer | 700.6 [M+H]+ | **264.2 (40)** / 682.6 (12) | 12.5 | 17:0 Glucosyl-Cer |
| 24:1 Hexosyl-Cer | 810.7 [M+H]+ | **264.2 (40)** / 792.7 (16) | 18.8 | 17:0 Glucosyl-Cer |
| 17:0 Lactosyl-Cer | 876.6 [M+H]+ | **264.3 (52)** / 534.5 (24) | 12.6 | - |
| 16:0 Lactosyl-Cer | 862.6 [M+H]+ | **264.3 (48)** / 520.5 (20) | 12.1 | 17:0 Lactosyl-Cer |
| 24:1 Lactosyl-Cer | 972.7 [M+H]+ | **264.3 (56)** / 630.7 (28) | 17.8 | 17:0 Lactosyl-Cer |

^a^ Quantifiers are given in bold. Collision energies (in eV) are shown in parentheses.

(*Cer, ceramide; dhCer, dihydroceramide; dhSM, dihydrosphingomyelin; dhSph, dihydrosphingosine; S1P, sphingosine-1-phosphate; SM, sphingomyelin; Sph, sphingosine*)

Table S3: Primers used for RT-qPCR

| **Name** | **Sequence 5‘-3‘** | **Reference** |
| --- | --- | --- |
| SPHK1 fwd | CCTGACCAACTGCACGCTAT | This study |
| SPHK1 rev | CTGAGCACAGAGAAGAGGCG | This study |
| SPHK2 fwd | AGCGTGGTAGCCACTTCAG | [3] |
| SPHK2 rv | GAGCAGTGTACCGATGCCA | [3] |
| SGPP1 fwd | CTGGTGTTCTCTAGTTTGCCTAAG | [4] |
| SGPP1 rev | GGTTGAAGTTGTCAATCAGGTCC | [4] |
| SGPL1 fwd | GATATCTTCCCAGGACTACG | [5] |
| SGPL1 rev | CATCATCTTCGTCAATGG | [5] |
| ASAH1 fwd | GGAGTTGCGTCGCCTTAGTC | This study |
| ASAH1 rev | CTGCAGTCCTCTGTCCACG | This study |
| UBC fwd | ATTTGGGTCGCGGTTCTTG | [6] |
| UBC rev | TGCCTTGACATTCTCGATGGT | [6] |
| YWHAZ fwd | ACTTTTGGTACATTGTGGCTTCAA | [6] |
| YWHAZ rev | CCGCCAGGACAAACCAGTAT | [6] |

Table S4: Antibodies, lipids and dyes used for this study.

| **Primary Antibodies** | | |
| --- | --- | --- |
| **Target** | **Source** | **Reference** |
| **Chlamydial HSP60 (A57-B9) (ms)** | **Santa Cruz** | **sc-57840** |
| **CERT (rb)** | **Abcam** | **ab72536** |
| **Secondary Antibodies** | | |
| **Target** | **Source** | **Reference** |
| **Goat anti-Mouse IgG (H+L) Cross-Adsorbed Secondary Antibody, Alexa Fluor 488** | **Invitrogen** | **A-11001** |
| **Goat anti-Mouse IgG (H+L) Highly Cross-Adsorbed Secondary Antibody, Alexa Fluor™ Plus 405** | **Invitrogen** | **A48255** |
| **Goat anti-Mouse IgG (H+L) Cross-Adsorbed Secondary Antibody, Alexa Fluor 555** | **Invitrogen** | **A-21422** |
| **Goat anti-Rabbit IgG (H+L) Cross-Adsorbed Secondary Antibody, Alexa Fluor 555** | **Invitrogen** | **A-21428** |
| **Dyes** | | |
|  | **Source** | **Reference** |
| **DAPI** | **Sigma Aldrich** | **D9542-5MG** |
| **BODIPY-FL-DBCO** | **Jena Biosciences** | **CLK-040-05** |
| **AlexaFluor^TM^ 488-DBCO** | **Jena Biosciences** | **CLK-1278** |
| **AlexaFluor^TM^ 546 azide** | **Atto-Tec** | **AD647N** |
| **Lipids** | | |
|  | **Source** | **Reference** |
| **ω-Azido-Sphingosine** | **Jürgen Seibel, Julius-Maximilians- University Würzburg, Germany** | **JF169 [7]** |
| **α-Amino-ω-Azido-C6-Ceramide** |  | **JF241 [8]** |
| **Trifunctional-Sphingomyelin1** |  | **[9]** |
| **Trifunctional-Sphingomyelin2** |  | **[9]** |

# **Supplemental Data Set 1 Legend**

**Supplemental Data Set 1: Overview of sphingolipid-associated genes with significantly changed expression during *Chlamydia* infection**. Genes detected as significantly changed (adj. p-values <0.05, regardless of log2 fold change) in the transcriptome 30 h p.i. (*Ctr* vs Mock) were assigned to the categories: s*phingolipid biosynthetic processes* (GO:0030148), *sphingolipid catabolic processes* (GO:0030149), *sphingolipid mediated signaling* (GO:0090520), *sphingolipid transfer activity* (GO:0120016) and *sphingolipid metabolic processes* (GO:0006665). Log2 foldchanges and adjusted p-values are listed. Gene annotations were downloaded from stringDB [10].

# **Supplemental references**

1. Ashburner, M., et al., *Gene Ontology: tool for the unification of biology.* Nature Genetics, 2000. **25**(1): p. 25-29.

2. Consortium, T.G.O., et al., *The Gene Ontology knowledgebase in 2023.* Genetics, 2023. **224**(1).

3. Fohmann, I., et al., *Sphingosine kinase 1/S1P receptor signaling axis is essential for cellular uptake of Neisseria meningitidis in brain endothelial cells.* PLOS Pathogens, 2023. **19**(11): p. e1011842.

4. Tantikanlayaporn, D., et al., *Sphingosine 1 Phosphate Modulates the Effect of Estrogen in Human Osteoblasts.* JBMR Plus, 2018. **2**(4): p. 217-226.

5. Prasad, R., et al., *Sphingosine-1-phosphate lyase mutations cause primary adrenal insufficiency and steroid-resistant nephrotic syndrome.* The Journal of Clinical Investigation, 2017. **127**(3): p. 942-953.

6. Vandesompele, J., et al., *Accurate normalization of real-time quantitative RT-PCR data by geometric averaging of multiple internal control genes.* Genome Biology, 2002. **3**(7): p. research0034.1.

7. Lang, J., et al., *Acid ceramidase of macrophages traps herpes simplex virus in multivesicular bodies and protects from severe disease.* Nature Communications, 2020. **11**(1): p. 1338.

8. Götz, R., et al., *Nanoscale imaging of bacterial infections by sphingolipid expansion microscopy.* Nature Communications, 2020. **11**(1): p. 6173.

9. Rühling, M., et al., *Trifunctional sphingomyelin derivatives enable nanoscale resolution of sphingomyelin turnover in physiological and infection processes via expansion microscopy.* Nature Communications, 2024. **15**(1): p. 7456.

10. Szklarczyk, D., et al., *The STRING database in 2023: protein-protein association networks and functional enrichment analyses for any sequenced genome of interest.* Nucleic Acids Res, 2023. **51**(D1): p. D638-d646.
